# Isolation and Characterization of New Phenolic Compounds with Estrogen Biosynthesis-Inhibiting and Antioxidation Activities from *Broussonetia papyrifera* Leaves

**DOI:** 10.1371/journal.pone.0094198

**Published:** 2014-04-08

**Authors:** Chunyan Yang, Fu Li, Baowen Du, Bin Chen, Fei Wang, Mingkui Wang

**Affiliations:** 1 Key Laboratory of Mountain Ecological Restoration and Bioresource Utilization & Ecological Restoration Biodiversity Conservation & Key Laboratory of Sichuan Province, Chengdu Institute of Biology, Chinese Academy of Sciences, Chengdu, China; 2 University of Chinese Academy of Sciences, Beijing, China; Islamic Azad University-Mashhad Branch, Mashhad, Iran, Iran (Republic of Islamic)

## Abstract

*Broussonetia papyrifera* leaves (BPL) as a traditional Chinese medicine are also used in livestock feed for stimulating reproduction, adipose tissue and muscle development; however, the mechanism of their action is still unknown. Through estrogen biosynthesis-guided fractionation in human ovarian granulosa-like KGN cells, five new phenolic glycosides, broussoside A–E(**1**–**5**), along with fifteen known dietary phenolic compounds, were isolated from the *n*-butanol extract of BPL, and their structures were elucidated on the basis of NMR spectra analysis and chemical evidence. New compounds **3**, **4**, **5** and the known compounds **9** and **10** were found to potently inhibit estrogen biosynthesis in KGN cells. In addition, compounds **9**, **17**, **18**, and **20** showed strong antioxidant activity against ABTS (2,2′-azino-bis(3-ethylbenzothiazoline-6-sulfonic acid) diammonium salt) and DPPH (1, 1′-diphenyl -2-picryl-hydrazyl radical) assays. These findings suggest that BPL may improve meat quality through the regulation of estrogen biosynthesis. Furthermore, they may be useful for the discovery of potential aromatase modulators from natural products. Finally, they could be considered as a new source for natural antioxidants.

## Introduction

Estrogen plays a vital role in the regulation of many biological processes, which is synthesized by aromatase in the body, the only enzyme in vertebrate that can catalyze the formation of estrogens by using androgens as substrates [1]. Synthetic aromatase inhibitors have been developed for the treatment of breast cancer; however, their clinical uses are severely limited by their side effects such as increased risk for osteoporosis and cardiovascular disease [2]. Natural products are considered a good source of aromatase inhibitors; however, only about 300 natural products have been evaluated for their effects on aromatase [3]. Estrogen is also known to affect animal reproduction, adipose tissue and muscle development [4]–[6]. In livestock husbandry, hormones such as estrogen are widely used to stimulate these processes, but they often lead to food safety issues caused by the residual hormones [5]. Thus, finding new natural aromatase modulators and feeding sources is a new option for estrogen-related diseases and livestock culturing.

Some phenolic compounds have been reported to show potent estrogen biosynthesis-inhibiting activity [7]. Besides, they have been also reported to have excellent antioxidant properties [8], [9]. Natural antioxidants can protect the human body from free radicals, retard the progress of many chronic diseases, and provide a barrier to the oxidative rancidity of lipids in food, cosmetics, and pharmaceutical materials [10]. Therefore, the isolation and characterization of natural antioxidants, with little or no side effects, for use in foods or medicinal materials is still necessary for the replacement of their synthetic counterparts whose safety has been questioned for a long time.

*Broussonetia papyrifera* (L.) Vent., which belongs to the family of *Moraceae*, and is also known as paper mulberry, is a deciduous tree or shrub widely spread in Asia and Pacific countries such as China, USA and Thailand with multiple functions that are extensively applied in the papermaking, livestock feeding, medicine, etc [11]–[13]. *B. papyrifera* has been used for the treatment of hernias, dysentery, tinea, and oedema in traditional Chinese medicine [14]. Its leaves are often made into medicinal tea in China because their extracts have shown antihepatotoxic, antioxidant [15], antifungal, and lens aldose reductase inhibitory activities. The leaves of *B. papyrifera* are also widely used in China to feed livestock for improving meat quality [16]. In addition to nutritional compounds, *B. papyrifera* is also rich in phenolic compounds [17]. Since the first report of the use of Broussonin A as a source of phytoalexins, researchers have focused on the isolation of the bioactive constituents from *B. papyrifera*. Its main bioactive constituents are phenolic compounds, diterpenoids and alkaloids [12]. However, the effect of phenolic glycosides from BPL on estrogenic biosynthesis and oxidation is still unknown. In this study, *B. papyrifera* leaves were investigated through bioactivity-guided isolation, and the effects of isolated compounds on estrogen biosynthesis and oxidation were evaluated.

## Materials and Methods

### Ethics Statement

The *B. papyrifera* leaves were collected from Chongqing, China, in June 2011 and identified by Professor Weikai Bao, Chengdu Institute of Biology, Chinese Academy of Sciences. No specific permits were required for the described field studies in Chongqing. The research sites are not privately owned or protected in any way and field studies did not involve endangered or protected species. Human ovarian granulosa-like KGN cells as described in reference [18] were kindly supplied by Prof. Yiming Mu, Chinese PLA General Hospital, Beijing, China. No specific permissions were required for these activities.

### General Procedures

2,2′-Azinobis(3-ethylbenzothiazoline-6-sulfonic acid) diammonium salt (ABTS), 2,2′-dipheny1-1-picrylhydrazyl (DPPH), potassium persulfate (K_2_S_2_O_8_), and all the chromatographic solvents (methanol and acetonitrile) were purchased from Sigma (St. Louis, USA). The TSKgel HW-40C was purchased from TOSOH Corporation. Sephadex LH-20 was purchased from GE Company. HPLC-grade water was prepared from a Milli-Q system (Millipore Laboratory, Bedford, MA). Column chromatography was carried out on silica gel (200–300 mesh) supplied by Qingdao Marine Chemical Co. Thin-layer chromatography (TLC) was carried on silica gel GF_254_-precoated plates with chloroform/methanol/water (6∶4∶1) and the spots were visualized by UV illumination (254 nm) and by spraying with 5% H_2_SO_4_ in ethanol followed by heating. NMR spectra were recorded in DMSO-d_6_ with a Bruker Avance 600 spectrometer for both ^1^H- and ^13^C-NMR. Coupling constants were expressed in Hertz and chemical shifts were given on a ppm scale with tetramethylsilane (TMS) as the internal standard. The spectroscopic data of ^1^H-NMR, ^13^C-NMR, HSQC, HMBC for broussoside A–E were shown in the Supporting Information (Figures S1–20 in File S1). Analytical high-performance liquid chromatography (HPLC) was carried out on a LabAlliance Series III with a model 201 (SSI) detector and Ultimate C_18_ column (250 mm×4.60 mm, 5 μm). Preparative HPLC was carried out on a P3000 with a UV3000 detector (Beijing ChuangXinTongHeng Science and Technology Co., Ltd) and Ultimate C_18_ column (250 mm×21.2 mm, 5 μm). Electrospray ionization mass spectrometry (ESI-MS) and High resolution electrospray ionization mass spectrometry (HR-ESI-MS) were measured on a Finnigan LCQ^DECA^ and Bruker Apex-III mass spectrometer respectively.

### Extraction and Isolation

**Figure 1:**
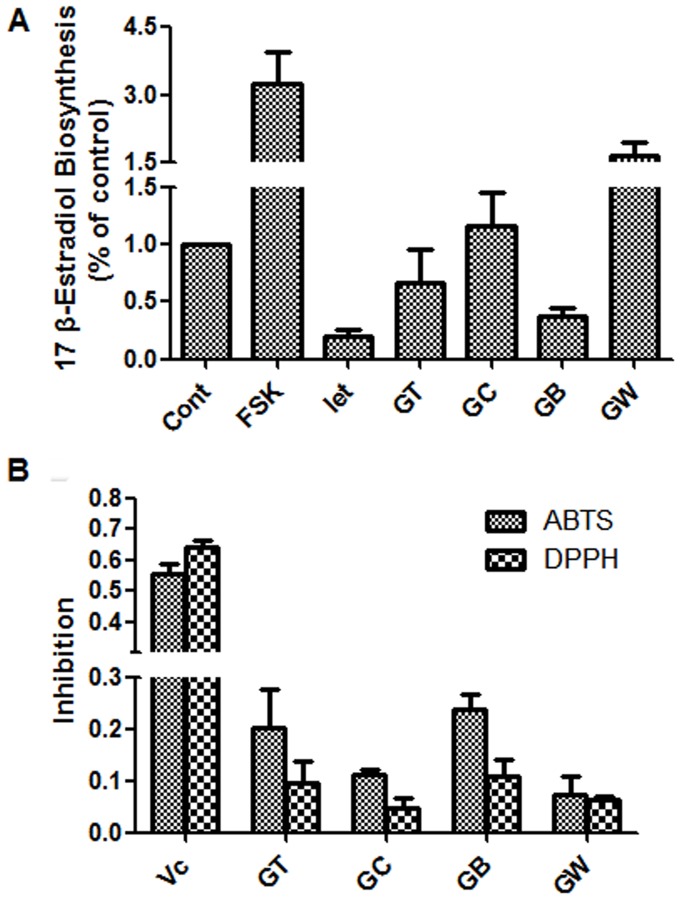
Estrogen biosynthesis and antioxidant activities of the crude samples from the BPL. (A) KGN cells were seeded in 24-well plates and pretreated with the crude samples at 50 μg/mL for 24 h. The cells were then supplemented with 10 nM testosterone for further 24 h, and 17β-estradiol in the culture medium was quantified using a magnetic particle-based 17β-estradiol ELISA. (B) ABTS and DPPH solutions were prepared daily and diluted to an absorbance of 0.70±0.02 at 734 nm (ABTS) and 517 nm (DPPH). After the crude samples (10 μg/mL) reacted with the ABTS radical solution for 10 min (DPPH radical solution for 30 min), the absorbance value (Ai) of ABTS at 734 nm (or Ai of DPPH at 517 nm) was measured, and the percentage inhibition was calculated. Cont., DMSO control; FSK, 10 μM Forskolin; Let, 1 μM letrozole; Vc, ascorbic acid; GT, methanol extract; GC, chloroform soluble extract; GB, *n*-butanol soluble extract; GW, aqueous extract.

Dried leaves from *B. papyrifera* (31 kg) were extracted with a 10-fold (w/v) volume of methanol by steeping for 3 days at room temperature three times, and then filtered. The clarified solvent was evaporated under reduced pressure to afford the methanol extract (GT; 6.04 kg). The extract then was suspended in distilled water (18000 mL) and partitioned successively with chloroform (6000 mL×4), and *n*-butanol (6000 mL×4) to yield the chloroform soluble fraction (GC; 2.01 kg), *n*-butanol soluble fraction (GB; 905.6 g) and aqueous fraction (GW; 3.12 kg). The GB extracts showed better radical-scavenging activity (**Figure 1**) than the other three extracts, and was thus subjected to further purification over a silica gel column (4 kg, 5×50 cm, 200–300 mesh, Qingdao Haiyang Chemicals, Qingdao China). A stepwise elution of chloroform–methanol, from 1∶0 to 2∶1 (water saturated), was done to yield nine fractions. Fraction 2 (23.32 g) was further purified by Toyopearl TSK HW-40C gel chromatography using a 3.5-cm–×–40-cm column, followed by preparative high performance liquid chromatography (PHPLC) to give compounds **6** (55.9 mg), **7** (24.1 mg), **8** (87.9 mg), and **9** (164.6 mg). Fraction 3 (13.37 g) was subjected to Sephadex LH-20 gel chromatography using a 4.0-cm–×–70.0-cm column, followed by PHPLC to obtain compounds **10** (19.3 mg), **11** (44.2 mg), and **12** (10.8 mg). Fraction 4 (7.34 g) was subjected to Sephadex LH-20 gel chromatography using a 4.0-cm–×–70.0-cm column, followed by PHPLC to get compound **13** (96.4 mg). Fraction 5 (16.41 g) was subjected to Sephadex LH-20 gel chromatography using a 4.0-cm–×–70.0 cm column, followed by PHPLC to get compounds **4** (24.2 mg), **14** (44.4 mg), and **15** (21.3 mg). Fraction 6 (18.66 g) was subjected to Sephadex LH-20 gel chromatography using a 4.0-cm–×–70.0-cm column, followed by PHPLC to get compound **16** (21.3 mg). Fraction 7 (24.13 g) was subjected to Sephadex LH-20 gel chromatography using a 4.0-cm–×–70.0-cm column, followed by PHPLC to get compounds **1** (12.8 mg), **2** (233.2 mg), **3** (44.6 mg), **5** (28.5 mg), **17** (22.6 mg), **18** (45.8 mg), and **19** (23.4 mg). Fraction 8 (11.17 g) was subjected to Sephadex LH-20 gel chromatography using a 4.0-cm–×–70.0-cm column, followed by PHPLC to get compound **20** (120.0 mg).

### Acid Hydrolysis of the New Compounds and Sugar Analysis

The new compounds **1**–**5** (2 mg) were added to a solution of concentrated HCl (0.5 mL), and H_2_O (1.5 mL)/dioxane (3 mL) and refluxed for 3 h. After dilution with H_2_O, the reaction mixture was extracted twice with ethyl acetate (EtOAc). The EtOAc layer of compounds **4** and **5** were then concentrated to dryness under reduced pressure. The residue was re-dissolved with methanol (2 mL) for an optical rotation measurement. The H_2_O layers of compounds **1**–**5** were neutralized with NaHCO_3_ and then concentrated to dryness under reduced pressure. The residue was re-dissolved with H_2_O (2 mL) for optical rotation measurement and TLC analysis.

### Cell Proliferation Assay

KGN cells as described in the reference [18] were seeded into 96-well plates (1.0×10^3^ cells/100 μL) with Dulbecco’s modified Eagle’s medium/nutrient mixture F-12 (DMEM/F-12) and were incubated at 37°C in a 5% CO_2_ humidified atmosphere. Compounds were then added to the cells, and the plates were incubated for a further 24 h. Alamar Blue reagent (10 μL/well) was added, and the fluorescence intensities were measured using a Verioskan Flash Multimode Reader with excitation at 544 nm and emission at 590 nm. Cytotoxicity was defined as the ratio of the fluorescence intensity in the test wells to that in the solvent control wells (0.1% dimethyl sulfoxide; DMSO). The assay was conducted in triplicate.

### Cell-based Estrogen Biosynthesis Assay

The cell-based estrogen biosynthesis assay was performed as previously described [19]. Briefly, KGN cells were seeded in 24-well plates overnight. The following day, the cells were replaced with serum-free DMEM/F-12 medium and pretreated with the test chemicals for 24 h. Testosterone (10 nM) was then added to each well, and the cells were incubated for a further 24 h. The culture media and cell lysates were collected and stored at –20°C. The 17β-estradiol in the culture medium was quantified using a 17β-estradiol magnetic particle-based enzyme-linked immunosorbent assay (ELISA) according to the manufacturer’s instructions. The optical density (OD) value was measured at 550 nm with the Verioskan Flash Multimode Reader (Thermo Scientific, Waltham, MA). The results, normalized to the total cellular protein content, were expressed as percentages of the control.

### ABTS and DPPH Radical-scavenging Assay

The antioxidant capacity of the test samples (the extracts and phenolic compounds) were measured by their ability to scavenge the ABTS radical cation and DPPH radical using previously reported methods [20], [21], with some modifications. l-Ascorbic acid was used as a positive control. Assays were performed in 96-well plates. The ABTS radical was prepared by reacting 10 mL of 2 mM ABTS water solution with 100 μL of 70 mM potassium persulfate, and the mixture was allowed to stand in dark at room temperature for 12 h before use. Prior to the assay, the solution was diluted in water to give an absorbance at 734 nm of 0.70±0.02 and was equilibrated at 25°C. The DPPH radical ethanol solution was obtained by dissolving 5 mg DPPH in 50 ml ethanol (EtOH) overnight in dark and then diluting to an absorbance of 0.70±0.02 at 517 nm. An EtOH solution (100 μL) of each sample (10–300 μg) was added to the ABTS (DPPH) radical ethanol solution (100 μL). After reacting with the ABTS radical solution for 10 min (DPPH radical solution for 30 min), the absorbance value (Ai) of ABTS at 734 nm (or Ai of DPPH at 517 nm) was measured using a Verioskan Flash Multimode Reader (Thermo Scientific; Waltham, MA, USA). The blank absorbance (A_0_) was measured using ethanol. The ABTS and DPPH radical solutions were prepared daily. The antioxidant activity was expressed as the percentage inhibition of the ABTS/DPPH radical and was determined by the following equation:
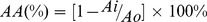



### Statistical Analysis

All experiments were carried out in triplicate. The inhibitory concentration providing 50% inhibitory capability (IC_50_) and the sample concentrations providing 50% scavenging capability (SC_50_) were obtained by fitting dose-response data to a four-parametric logistic nonlinear regression model using GraphPad Prism 5.0 software (GraphPad, La Jolla, CA).

## Results

### Estrogen Biosynthesis and Antioxidant Activities of the Extracts from BPL

To investigate whether the beneficial effects of BPL are because of their role in estrogen biosynthesis, we conducted a bioactivity-guided isolation procedure using human ovarian granulosa-like KGN cells. Forskolin, an adenylate cyclase agonist, increases intracellular cAMP and the expression of aromatase by activating the protein kinase A/CREB pathway in ovaries; we used it here as a positive control for an estrogen biosynthesis agonist [22]. Letrozole is a potent non-steroidal aromatase inhibitor registered for clinical use, and we used it here as a positive control for an estrogen biosynthesis antagonist [23]. As shown in **Figure 1A**, forskolin promoted the production of 17β–estradiol in KGN cells, whereas letrozole significantly inhibited it, the same as previously reported [24]. The methanol extract (GT) and the *n*-butanol-soluble extract (GB) of the BPL showed the most potent estrogen biosynthesis-inhibiting activity at 50 μg/mL, whereas the CHCl_3_-soluble extract (GC) and the water-soluble extract (GW) had no such effect.

To investigate the effect of BPL on oxidation, we evaluated the antioxidant activity of the BPL extracts. As shown in **Figure 1B**, the *n*-butanol-soluble extract (GB) showed the most potent antioxidant activity (ca. 23.9% and 11.0% inhibition against ABTS and DPPH at 10 μg/mL, respectively). Therefore, the *n*-butanol-soluble extract (GB) was further purified on a silica gel column to obtain 8 subfractions. Final purifications were performed by HPLC to give the following twenty bioactive constituents (**1**–**20**).

### Structure Characterization

**Figure 2:**
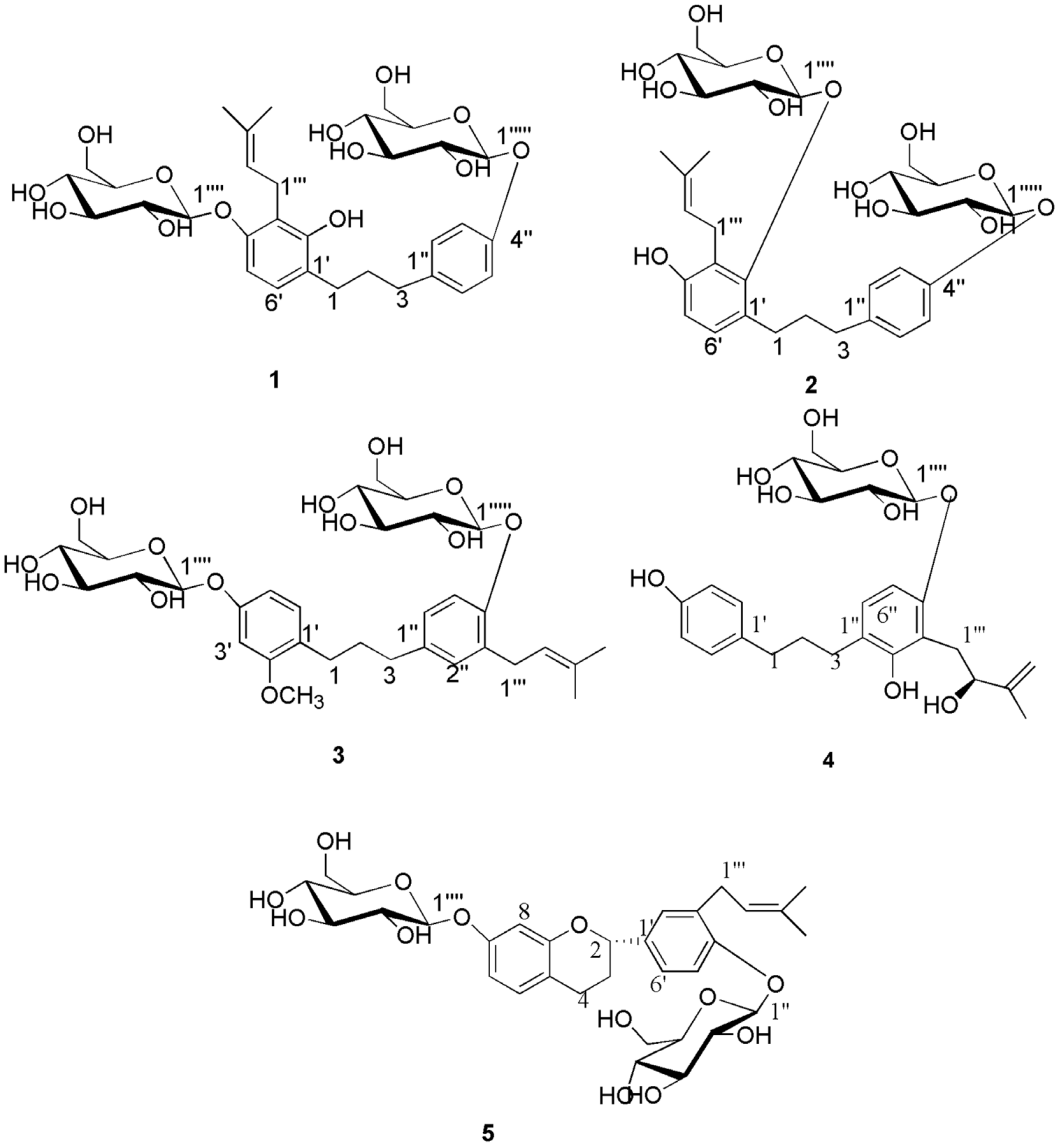
Chemical structures of the new compounds extracted from the BPL.

**Figure 3:**
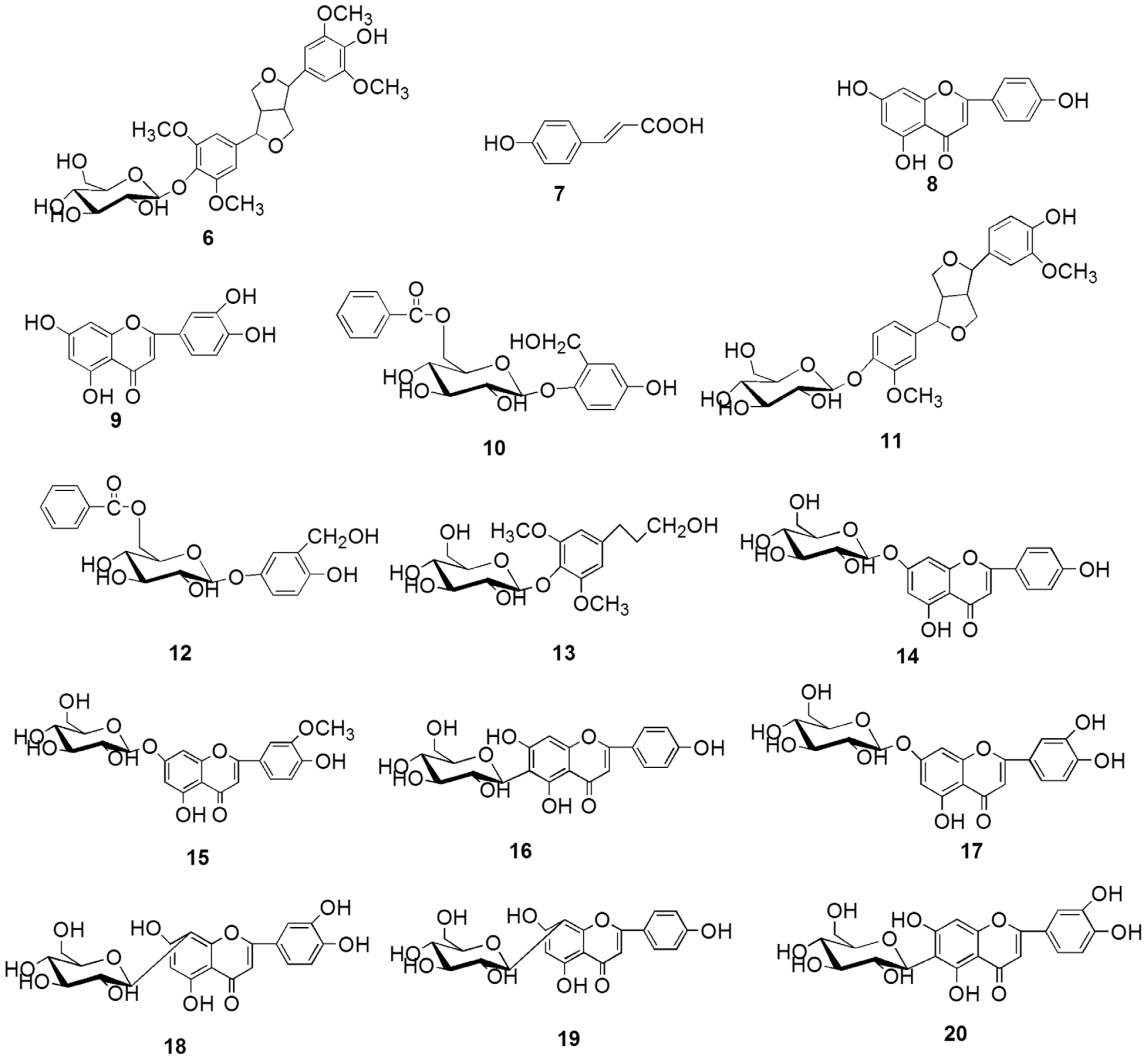
Chemical structures of the known compounds extracted from the BPL.

Bioassay-guided fractionation and purification of GT from BPL has afforded five new phenolic compounds: broussoside A (**1**), broussoside B (**2**), broussoside C (**3**), broussoside D (**4**), broussoside E (**5**), and their chemical structures were illustrated in **Figure 2**. Fifteen known dietary phenolic compounds (**Figure 3**) were also isolated and identified as syringaresinol-4′-*O*-*β*-D-glucoside (**6**) [25], *p*-coumaric acid (**7**) [26], [27], apigenin (**8**) [28], luteolin (**9**) [28], poliothyrsoside (**10**) [29], pinoresinol-4′-*O*-*β*-D-glucopyranoside (**11**) [30], [31], flacourtin (**12**) [31], dihydrosyringin (**13**) [32],apigenin-7-*O*-*β* -D-glucoside (**14**) [33], chrysoriol-7-*O*-*β*-D-glucoside (**15**) [34], isovitexin (**16**) [35], luteoloside (**17**) [33], orientin (**18**) [36]–[38], vitexin (**19**) [35], isoorientin (**20**) [38], [39] on the basis of comparison of their NMR data with those reported in the literature. With the exception of apigenin (**8**), luteolin (**9**), apigenin-7-*O*- *β*-D-glucoside (**14**), chrysoriol-7-*O*-*β*-D-Glucoside (**15**), luteoloside (**17**), vitexin (**19**), to the best of our knowledge, this is the first instance that these dietary phenolic compounds have been isolated from the genus *Broussonetia*.

**Figure 4:**
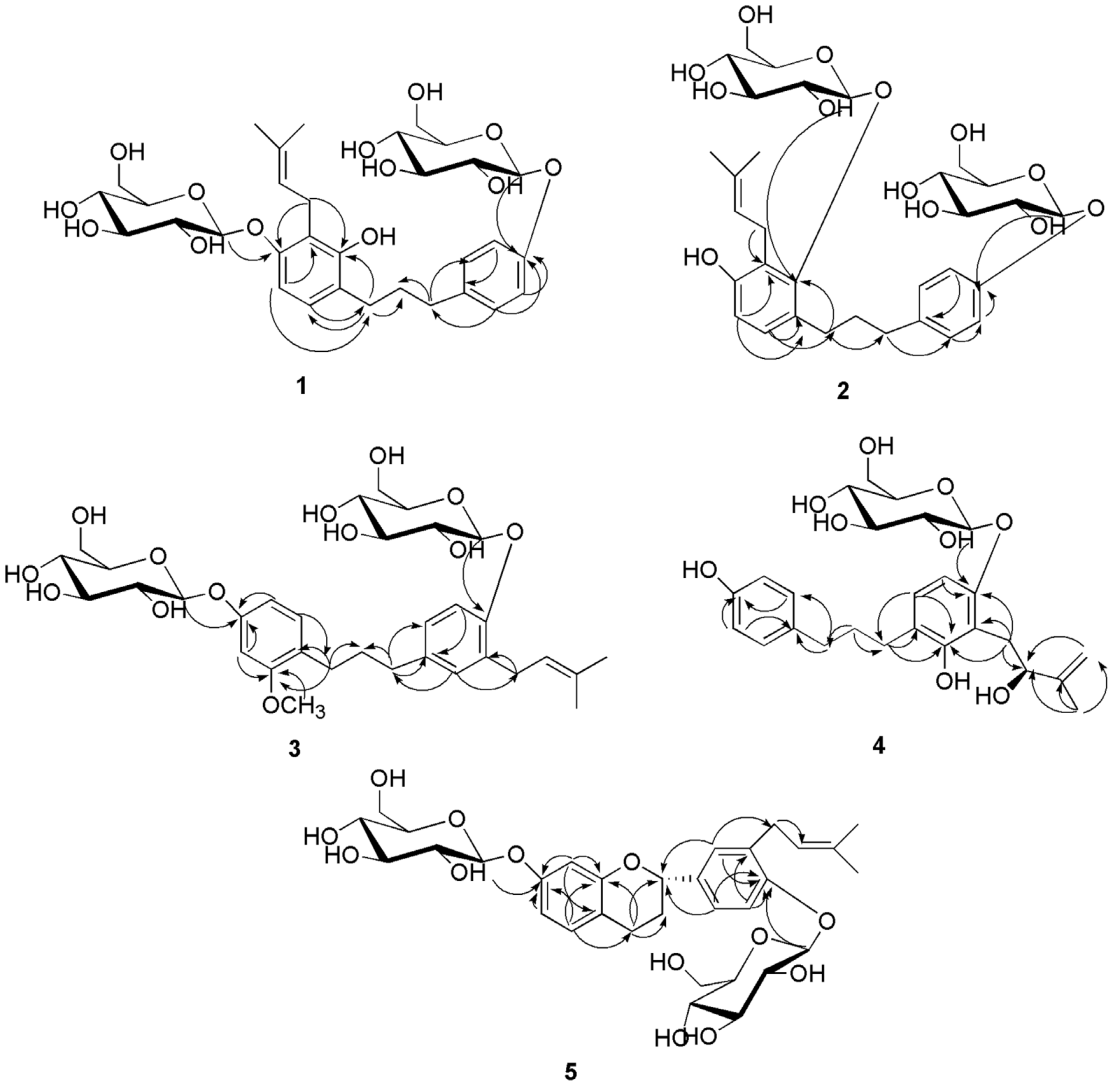
The key HMBC (H→C) correlations of the new compounds.

Compound **1** was obtained as a white powder having the molecular formula C_32_H_44_O_13_ and 11 degrees of unsaturation, as established by HR-ESI-MS [M+Na]^+^ ion at *m/z* 659.2689 (calcd 659.2674). The ^1^H-NMR data (**Table 1**) of **1** showed six aromatic proton signals at δ_H_ 6.54 (d, 1H, J = 8.4 Hz, H-5′), 6.79 (d, 1H, J = 8.4 Hz, H-6′), 7.10 (d, 2H, J = 9.0 Hz, H-2″, 6″), and 6.93 (d, 2H, J = 9.0 Hz, H-3″, 5″), revealing the presence of two AB system aromatic rings. In the aliphatic region, the signals coupled to each other at δ_H_ 1.75 (2H, multiplet, H-2), 2.55 (2H, multiplet, H-3) and 2.51 (2H, multiplet, H-1) suggested the presence of a 1, 3-diphenyl-substituted propane unit [40]. In addition, characteristic signals were observed for a prenyl group at δ_H_ 3.43 (1H, m, H-1″′), 3.22 (1H, m, H-1″′), 5.17 (1H, t, J = 7.2 Hz, H-2″′), and 1.60 (3H, singlet, H-4″′), 1.73 (3H, singlet, H-5″′), and two sugar units were identified on the basis of the presence of two anomeric proton signals at δ_H_ 4.70 (d, J = 7.0 Hz, H-1″″), and 4.79 (d, J = 7.8 Hz, H-1″″′). A hydroxyl group at δ_H_ 8.07 (s) was also observed in the ^1^H-NMR spectrum. The ^13^C-NMR spectrum (**Table 2**) and HSQC spectra of **1** showed 32 carbon signals, which were attributed to two sugar moieties, a 1,3-diphenyl propane skeleton and a prenyl group. The ^1^H and ^13^C-NMR spectra (**Table 1** and **Table 2**) were similar to those of 1-(2,4-dihydroxy-3-prenylphenyl) -3-(4-hydroxyphenyl) propane [40], a 1,3-diphenyl propane, except for the absence of two sugar groups. The HMBC correlation (**Figure 4**) of the peak at δ_H_ (3.43, m, H-1″′), (3.22, m, H-1″′) with C-2′ (δ_C_ 152.6), C-4′ (δ_C_ 154.5) unveiled the location of the prenyl moiety. The correlations observed in the HMBC experiment between the glucosyl anomeric proton (H-1″″) and C-4′, between the glucosyl anomeric proton (H-1″″′) and C-4″, established that the sugar units were located at C-4′, 4″ of the aglycone. The anomeric proton coupling constants indicated the presence of two β-glucosyl units [41]. The sugar moiety was determined as d -glucose by TLC analysis and measuring the optical rotation ([α]
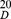
+51.2° (c 0.05, H_2_O)) of the acid hydrolysis solution of **1**. Therefore, the structure of compound **1** was determined as (2′-hydroxy- 3′-prenyl-1,3-diphenylpropane) -4′, 4″-di-*O*-*β*- d -glucopyranoside and this compound has been assigned the name broussoside A.

**Table 1:** 1H-NMR spectral data °f the newly isolated compounds 1, 2, and 3 (600 MHz in DMSO-d6, δ in ppm, J in Hz).

position	1	2	3
1	2.51 (m)	2.66 (m)	2.46 (m)
2	1.75 (m)	1.78 (m), 1.70 (m)	1.73 (m)
3	2.55 (m)	2.53 (m)	2.46 (m)
3′			6.65 (d, 2.4)
5′	6.54 (d, 8.4)	6.52 (d, 7.8)	6.53 (dd, 2.4, 8.4)
6′	6.79 (d, 8.4)	6.79 (d, 7.8)	6.98 (d, 8.4)
2″	7.10 (d, 9.0)	7.11 (d, 8.4)	6.89 (d, 1.8)
3″	6.93 (d, 9.0)	6.93 (d, 8.4)	
5″	6.93 (d, 9.0)	6.93 (d, 8.4)	6.97 (d, 8.4)
6″	7.10 (d, 9.0)	7.11 (d, 8.4)	6.92 (dd, 1.8, 8.4)
1″′	3.22 (m), 3.43 (m)	3.25 (brd, 8.4), 3.55 (dd, 5.4, 12.0)	3.24 (m), 3.34 (m)
2″′	5.17 (t, 7.2)	5.16 (t, 6.6)	5.29 (t, 7.8)
4″′	1.60 (s)	1.60 (s)	1.65 (s)
5″′	1.73 (s)	1.70 (s)	1.67 (s)
1″″	4.70 (d, 7.2)	4.49 (d, 7.2)	4.80 (d, 7.2)
2″″	3.24 (m)	3.21 (m)	3.24 (m)
3″″	3.29 (m)	3.28 (m)	3.27 (m)
4″″	3.16 (m)	3.14 (m)	3.14 (m)
5″″	3.29 (m)	3.28 (m)	3.27 (m)
6″″	3.46 (m), 3.69 (dd, 4.2, 10.8)	3.46 (m), 3.69 (brd, 10.8)	3.45 (m), 3.69 (m)
1″″′	4.79 (d, 7.8)	4.79 (d, 7.8)	4.73 (d, 7.2)
2″″′	3.24 (m)	3.21 (m)	3.24 (m)
3″″′	3.29 (m) 3.29 (m)	3.28 (m)	3.27 (m)
4″″′	3.16 (m)	3.14 (m)	3.14 (m)
5″″′	3.29 (m)	3.28 (m)	3.27 (m)
6″″′	3.46 (m), 3.69 (dd, 4.2, 10.8)	3.42 (m), 3.62 (brd, 10.8)	3.45 (m), 3.69 (m)
-OCH_3_			3.73 (s)
-OH	8.07 (s)	9.04 (s)	

**Table 2:** ^13^C-NMR spectral data of the newly isolated compounds 1, 2, 3, 4, and 5 (150 MHz in DMSO-d_6_, δ in ppm).

Position	1	2	3	4	Position	5
1	29.2	28.7	28.7	29.3	2	76.6
2	31.8	32.6	31.5	31.8	3	29.0
3	34.2	34.5	34.3	34.2	4	23.7
1′	118.1	122.0	123.3	132.3	4a	115.3
2′	152.6	153.4	157.8	129.0	5	129.8
3′	122.7	125.8	101.5	115.0	6	109.0
4′	154.5	154.1	157.0	155.2	7	156.7
5′	106.4	111.2	107.5	115.0	8	104.0
6′	126.6	126.7	130.1	129.0	8a	155.3
1″	135.5	135.8	135.4	123.1	1′	130.2
2″	129.0	129.0	129.1	154.2	2′	127.1
3″	116.1	116.1	129.7	115.9	3′	134.6
4″	155.5	155.5	153.5	154.7	4′	154.8
5″	116.1	116.1	115.0	106.6	5′	114.9
6″	129.0	129.0	126.5	127.4	6′	124.8
1″′	22.6	23.2	28.2	30.1	1″	101.3
2″′	123.6	124.3	123.1	75.1	2″	73.5
3″′	129.5	129.2	131.3	147.7	3″	76.7
4″′	25.6	25.5	25.7	109.4	4″	69.8
5″′	17.8	18.0	17.8	18.4	5″	77.0
1″″	101.4	104.6	100.4	101.6	6″	60.8
2″″	73.6	73.4	73.4	73.6	1″′	28.1
3″″	76.6	76.6	76.8	76.9	2″′	122.7
4″″	69.9	69.8	70.0	69.8	3″′	131.5
5″″	77.0	76.9	77.0	77.0	4″′	25.6
6″″	60.8	60.8	60.9	60.8	5″′	17.7
1″″′	100.6	100.7	100.8		1″″	100.7
2″″′	73.3	74.1	73.6		2″″	73.2
3″″′	76.6	76.6	76.9		3″″	76.8
4″″′	69.8	70.1	70.1		4″″	69.7
5″″′	77.0	77.0	77.2		5″″	76.9
6″″′	60.7	61.2	60.9		6″″	60.7
-OCH_3_			55.4			

Compound **2** was obtained as a white paste with the molecular formula C_32_H_44_O_13_ and 11 degrees of unsaturation, as established by HR-ESI-MS [M+Na]^+^ ion at *m/z* 659.2677 (calcd 659.2674). The NMR data (**Table 1** and **Table 2**) of compounds **2** and **1** are very similar. The only differences were one d-glucopyranosyl location (**Figure 2**) and the chemical shift of the hydroxyl group in the ^1^H-NMR spectrum. A hydroxyl group at δ_H_ 9.04 (s) was observed in the ^1^H-NMR spectrum instead of the chemical shift at δ_H_ 8.07 (s) in compound **1**. Two sugar units were identified by the presence of anomeric proton signals at δ_H_ 4.49 (d, J = 7.2 Hz, H-1″″), and 4.79 (d, J = 7.8 Hz, H-1″″′). The HMBC spectrum (**Figure 4**) of **2** showed a correlation between the sugars and the aglycone, H-1″″ (δ_H_ 4.70)/C-2′ (δ_C_ 153.4), and H-1″″′ (δ_H_ 4.79)/C-4″ (δ_C_ 155.5). These correlations indicated that the sugars are located at C-2′ and C- 4″ of the aglycone instead of the position of C-4′ and C- 4″ in compound **1**. The anomeric proton coupling constants indicated the presence of two β-glucosyl units [41]. The sugar moiety was determined as d-glucose by TLC analysis and the optical rotation ([α]
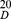
+51.8° (c 0.06, H_2_O)) of the acid hydrolysis solution of **2**. Based on the above evidence, compound **2** has been assigned as (4′-hydroxy-3′-prenyl-1,3-diphenylpropane)-2′,4″-di-*O*-*β*-d-diglucopyranoside and named broussoside B.

Compound **3** was obtained as a white paste with the molecular formula C_33_H_46_O_13_ and 11 degrees of unsaturation, as established by HR-ESI-MS [M+Na]^+^ ion at *m/z* 673.2851 (calcd 673.2831). To date, only one reported compound is somewhat similar to this compound, with the difference in the absence of two sugar groups (**Figure 2**). The correlations observed in the HMBC experiment (**Figure 4**) H-1″″/C-4′, H-1″″′/C-4″, established that the locations of the sugar units were at C-4′ and 4″. The anomeric proton coupling constants indicated the presence of two β-glucosyl units [41]. The sugar moiety was determined as d-glucose by TLC analysis and optical rotation ([α]
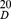
+50.9° (c 0.06, H_2_O)) of the acid hydrolysis solution of **3**. Therefore, the structure of compound **3** was determined to be (2′-methoxy-3″-prenyl-1,3-diphenylpropane)-4′,4″-di-*O*-*β*-d-glucopyranoside, and this new compound was assigned the name broussoside C.

Compound **4** was obtained as a white paste with the molecular formula C_26_H_34_O_9_ and 10 degrees of unsaturation, as established by HR-ESI-MS [M+Na]^+^ ion at *m/z* 513.2103 (calcd 513.2095). The position of the 2-hydroxyl-3-methylbut-3-enyl group was assigned to C-3″ from the correlation between the H-1″′ (δ_H_ 2.74, 2.98) (**Table 3**) and C-3″ (δc 115.9) (**Table 2**) and the correlation between the H-1″′ (δ_H_ 2.74, 2.98) and C-2″ (δ_C_ 154.2), C-4″ (δ_C_ 154.7) in the HMBC spectrum (**Figure 4**). The correlation observed in the HMBC experiment (**Figure 4**) between the glucosyl anomeric proton (H-1″′) and C-4″ established the position of the sugar unit. The anomeric proton coupling constants indicated the presence of a β-glucosyl units [41]. The sugar moiety was determined as d-glucose by TLC analysis and optical rotation ([α]
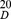
+51.7° (c 0.04, H_2_O)) of the acid hydrolysis solution of **4**. The absolute configuration at C-2″′ was confirmed as *S* using the specific rotation data ([α]
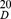
−2.5° (c 0.07, MeOH)) and comparing with the literature values for the compound Glepidotin C [42]. Therefore, the structure of **4** was determined as [4′,2″-dihydroxyl-3″-((2*S*)-2-hydroxyl-3-methylbut-3-enyl)-1,3-diphenylpropane]-4″-*O*-*β*-d-glucopyranoside, and this new compound was assigned the name broussoside D.

**Table 3:** 1H-NMR spectral data of the newly isolated compounds 4 and 5 (600 MHz in DMSO-d6, δ in ppm, J in Hz).

Position	4	Position	5
1	2.46 (m)	2	4.98 (dd, 1.8, 9.6)
2	1.73 (m)	3	1.95 (m), 2.05 (m)
3	2.49 (m)	4	2.62 (dt, 4.2, 16.2), 2.83(m)
2′	6.96 (d, 8.4)	5	6.96 (d, 8.4)
3′	6.65 (d, 8.4)	6	6.52 (dd, 2.4, 8.4)
5′	6.65 (d, 8.4)	8	6.45 (d, 2.4)
6′	6.96 (d, 8.4)	2′	7.13 (d, 1.8)
5″	6.59 (d, 8.4)	5′	7.06 (d, 8.4)
6″	6.83 (d, 8.4)	6′	7.16 (dd, 1.8, 8.4)
1″′	2.74 (dd, 9.0, 14.4), 2.98 (dd, 3.0, 14.4)	1″	4.77 (d, 7.8)
2″′	4.21 (dd, 1.2, 7.8)	2″	3.21 (m)
4″′	4.72 (brs), 4.92 (brs)	3″	3.26 (m)
5″′	1.77 (s)	4″	3.14 (m)
1″″	4.70 (d, 7.2)	5″	3.26 (m)
2″″	3.21 (m)	6″	3.44 (m), 3.67 (m)
3″″	3.23 (m)	1″′	3.27 (m), 3.37 (m)
4″″	3.13 (m)	2″′	5.29 (brt, 7.2)
5″″	3.23 (m)	4″′	1.65 (s)
6″″	3.46 (m), 3.69 (brd, 10.2)	5″′	1.66 (s)
		1″″	4.76 (d, 7.8)
		2″″	3.21 (m)
		3″″	3.26 (m)
		4″″	3.14 (m)
		5″″	3.26 (m)
		6″″	3.44 (m), 3.67 (m)

Compound **5** was recovered as a white paste with the molecular formula C_32_H_42_O_13_ and 12 degrees of unsaturation, as established by HR-ESI-MS [M+Na]^+^ ion at *m/z* 657.2533 (calcd 657.2518). To date, the ^1^H and ^13^C-NMR spectra of the aglycone were similar to those of (2*S*)-7,4′-dihydroxy-3′-prenylflavan [40], except for the absence of two sugar groups (**Figure 2**). The correlations observed in the HMBC experiment between the glucosyl anomeric proton (H-1″) and C-4′, between the glucosyl anomeric proton (H-1″″) and C-7, established the location of the sugar units. The anomeric proton coupling constants indicated the presence of two β-glucosyl units [41]. The sugar moiety was determined as d-glucose by TLC analysis and optical rotation ([α]
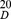
+52.3° (c 0.05, H_2_O)) of the acid hydrolysis solution of **5**. The absolute configuration at C-2 was confirmed as *S* using the specific rotation data ([α]
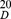
−4.7° (c 0.04, MeOH)) and comparing the data with the literature values for this flavan. Accordingly, the structure of the new compound **5** was assigned as [(2*S*)-3′-prenylflavan]-7,4′ -di-*O*-*β*-d-glucopyranoside, and this compound was assigned the name broussoside E.

### Effect of Isolated Compounds from BPL on Estrogen Biosynthesis in Human Ovarian Granulose-like KGN Cells

**Figure 5:**
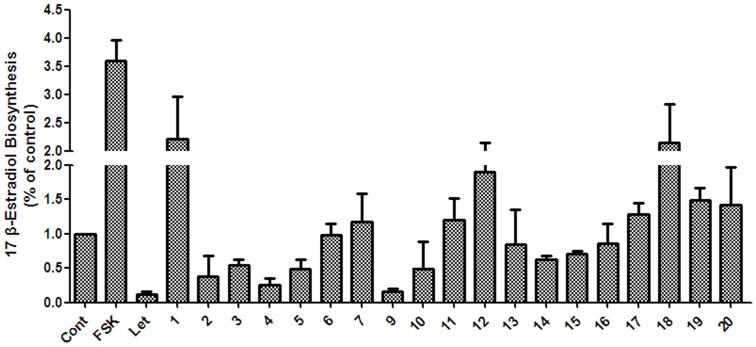
The estrogen biosynthesis activity of the isolated compounds from the BPL. KGN cells were seeded in 24-well plates and pretreated with test chemicals at 50 μM for 24 h. The cells were then supplemented with 10 nM testosterone for further 24 h, and 17β-estradiol in the culture medium was quantified using a magnetic particle-based 17β-estradiol ELISA. Cont., DMSO control; FSK, 10 μM Forskolin; Let, 1 μM letrozole.

All the isolated compounds were tested for their effect on estrogen biosynthesis activity (**Figure 5**). First, the cytotoxicity of the phenolic compounds (compounds **1–20**) at 50 μM against human ovarian granulosa KGN cells was evaluated. The results showed that only one compound, apigenin (**8**), was cytotoxic, whereas the other compounds displayed no cytotoxicity on the KGN cells (data not shown). Next, the isolated compounds (except for apigenin) were examined for their effects on estrogen biosynthesis in KGN cells at 50 μM. As shown in **Figure 5**, compounds **2**, **3**, **4**, **5**, **9** and **10** exhibited potent estrogen biosynthesis-inhibiting activity. Compounds **2**, **3**, **4**, **5**, **9**, and **10** were further subjected to IC_50_ testing. As seen from **Table 4**, the IC_50_ values of **2**, **3**, **4**, **5**, **9** and **10** were determined to be 45.02, 28.90, 20.20, 20.75, 1.32, and 11.89 μmol/L, respectively.

**Table 4:** The estrogen biosynthesis activity of compounds 2–5, 9 and 10.

Compound	IC_50_ a (μM)
**2**	45.02±4.38
**3**	28.90±0.84
**4**	20.20±0.11
**5**	20.75±1.96
**9**	1.32±2.13
**10**	11.89±3.28

a The IC_50_ value of each compound was defined as the concentration (μM) that caused 50% inhibition of estrogen biosynthesis in KGN cells.

### Effect of Isolated Compounds from BPL on Antioxidation

**Figure 6:**
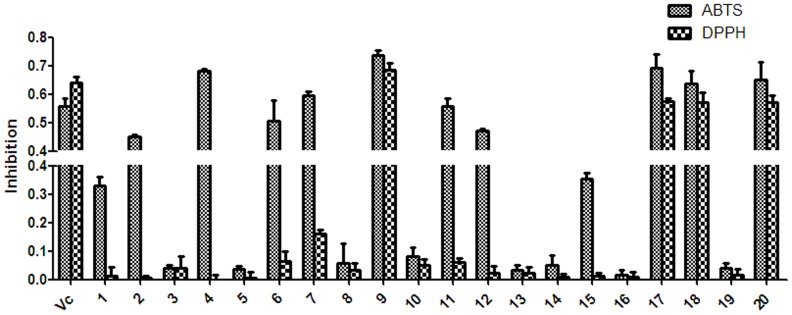
The radical-scavenging activity of the isolated compounds from the BPL. ABTS and DPPH solutions were prepared daily and diluted to an absorbance of 0.70±0.02 at 734 nm (ABTS) and 517 nm (DPPH). After the test chemicals (10 μg/mL) reacted with the ABTS radical solution for 10 min (DPPH radical solution for 30 min), the absorbance value (Ai) of ABTS at 734 nm (or Ai of DPPH at 517 nm) was measured, and the percentage inhibition was calculated. Vc, ascorbic acid.

To investigate the beneficial effects of BPL on oxidation, we evaluated the antioxidant activity of the isolated compounds from BPL (**Figure 6**). Twenty compounds, **1**–**20**, were evaluated for their antioxidant activity by measuring their ability to scavenge ABTS and DPPH free radicals. The SC_50_ values are given in **Table 5**. Among all of the compounds tested, **2**, **4**, **6**, **9**, **11**, **17**, **18** and **20** exhibited potent ABTS radical-scavenging activity, with SC_50_ values of 16.64, 11.8, 18.74, 21.22, 17.4, 14.06, 16.96, and 14.53 μmol/L, respectively. Four compounds, luteolin (**9**), luteoloside (**17**), orientin (**18**), and isoorientin (**20**) showed very strong DPPH radical-scavenging activity, with SC_50_ values of 19.72, 19.67, 18.86 and 19.33 μmol/L, respectively. l-Ascorbic acid was used as a positive control, with an SC_50_ value of 21.76 μmol/L against ABTS and 24.58 μmol/L against DPPH. The results indicated that the phenolic compounds with phenolic hydroxyl groups were antioxidants, consistent with the findings from a previous study [43]. Based on their SC_50_ values, the ABTS radical-scavenging activity of the compounds in descending order was as follows: **4>17>20>2>18>11>6**>**9**> l-ascorbic acid (reference) >**1**>**15**>**7>12>10>14>16>19>5>3>8>13**. While the descending order of the DPPH radical-scavenging activity of the compounds was as follows: **18>20>17>9**> l-ascorbic acid (reference) >**11>7**.

**Table 5:** Radical-scavenging activity of the compounds isolated from BPL.

Compound	MW	SC_50_ a values (μM)	
		ABTS	DPPH
**1**	636	27.23±0.21	>500
**2**	636	16.64±2.06	>500
**3**	650	177.98±0.29	>500
**4**	490	11.80±0.11	>500
**5**	634	162.54±0.14	>500
**6**	580	18.74±0.25	>500
**7**	164	43.05±0.08	200±2.11
**8**	270	293.63±0.16	>500
**9**	286	21.22±0.17	19.72±0.11
**10**	406	69.38±0.12	>500
**11**	520	17.4±1.10	170±0.18
**12**	406	43.57±0.16	>500
**13**	374	>500	>500
**14**	432	130.42±2.07	>500
**15**	462	32.9±1.07	>500
**16**	432	136.6±0.12	>500
**17**	448	14.06±0.08	19.67±0.32
**18**	448	16.96±0.22	18.86±2.31
**19**	432	137.94±2.15	>500
**20**	448	14.53±0.18	19.33±0.42
Ascorbic acid b	176	21.76±0.14	24.58±0.19

a The SC_50_ value of each compound was defined as the concentration (μM) that caused 50% scavenging capability of ABTS and DPPH.

b Ascorbic acid was used as a positive control.

## Discussion

In recent decades, the incidence of breast cancer has increased worldwide, and it is the main cause of cancer mortality and morbidity in women [44]. Overexposure to environmental estrogenic compounds and the western lifestyle have contributed to this increased incidence [19]. Inhibitors for the estrogen receptor and aromatase have been developed for the prevention or therapy of hormone-dependent breast cancer in postmenopausal women, and aromatase inhibitors show superior efficacy to the conventional antiestrogen receptor drugs such as tamoxifen [3]. In this study, we found that extracts from BPL were able to modulate the biosynthesis of estrogen, making it a possible beneficial health food.

Estrogen also plays an important role in the reproduction, muscle and adipose tissue formation [6]–[8]. BPL are used for feeding livestocks to improve the quality of the meat; however, the mechanism underlying this is still unknown. This study revealed that BPL extracts and the isolated natural compounds can modulate estrogen biosynthesis, suggesting that BPL may improve the meat quality by altering the endogenous estrogen levels. In addition, *B. papyrifera* is widely distributed in Asia and Pacific countries such as China, USA and Thailand, and is easily obtained in large quantities [13]–[15]. Thus, BPL can be developed as a natural supplement for feed in livestock husbandry to decrease the use of synthetic hormones.

In this study, we isolated several novel compounds. The new compounds **2**, **3**, **4**, **5** and the known compounds **9**, **10** exhibited estrogen biosynthesis-inhibiting activity, whereas, compounds **1**, **7**, **12**, **18**, **19** and **20** showed estrogen biosynthesis-promoting activity. Apigenin (**8**) and luteolin (**9**) were previously reported to potently inhibit aromatase activity [19]. Compounds **2** and **9** also inhibited estrogen biosynthesis and were obtained in large quantities. These two compounds may, therefore, make a large contribution to the strong inhibition of estrogen biosynthesis by the *n*-butanol extract of BPL, despite some compounds having an estrogen biosynthesis-promoting effect. To the best of our knowledge, this is the first time that compounds **3**, **4**, **5**, and **10** have been reported to inhibit estrogen biosynthesis, making them suitable for further investigation as new estrogen biosynthesis inhibitors for the prevention of breast cancer.

Free radicals are responsible for food decay and cause oxidative damages to biological systems [45]. According to the source and substrate attack mechanism of free radicals, antioxidants are divided into three categories: enzyme inhibitors, metal chelators, and radical scavengers. The former two prevent the generation of radicals indirectly, while the latter one scavenges radicals directly. Owing to their direct nature, the last category of radical-scavenging antioxidants has received much attention [46]. Therefore, the antioxidant capability of the extracts, fractions, and isolated compounds from the BPL was examined by ABTS and DPPH free radical assays. The compounds luteolin (**9**), luteoloside (**17**), orientin (**18**), and isoorientin (**20**) showed very potent ABTS/DPPH radical-scavenging activity. From a structural viewpoint, the four natural antioxidants were luteolin and luteolin derivatives with two vicinal phenolic hydroxyls on the B ring. These results suggest that antioxidant activity is determined not only by the number of phenolic hydroxyl groups but also by the position on the aromatic rings, which is consistent with the findings of a previous study [43]. Oxidative stress is responsible for many chronic diseases such as cancer and cardiovascular disorders [47]. The finding in this study that luteolin (**9**), luteoloside (**17**), orientin (**18**), and isoorientin (**20**) exhibit potent antioxidative activity not only explains the beneficial effects of BPL on human health, but also validates them as potential new food antioxidant supplements.

In conclusion, through estrogen biosynthesis-guided fractionation in human ovarian granulosa-like KGN cells, five new phenolic glycosides, together with fifteen known dietary phenolic compounds, were isolated from the *n*-butanol extract of BPL. BPL are used in livestock feed for stimulating reproduction, adipose tissue and muscle development; however, the mechanism is still unknown. The finding that the newly identified compounds **3**, **4**, and **5** and the known compounds **9** and **10** inhibit estrogen biosynthesis in KGN cells indicated that BPL may improve meat quality through the modulation of the endogenous estrogen levels. The finding that compounds **9**, **17**, **18**, and **20** showed potent antioxidant activity also contributes to the explanation of the beneficial effects of BPL on human health and validates them as potential new food antioxidant supplements.

## Supporting Information

File S1 Figures S1–S4, the spectroscopic data of the ^1^H-NMR, ^13^C-NMR, HSQC, HMBC for broussoside A. Figures S5–S8, the spectroscopic data of the ^1^H-NMR, ^13^C-NMR, HSQC, HMBC for broussoside B. Figures S9–S12, the spectroscopic data of the ^1^H-NMR, ^13^C-NMR, HSQC, HMBC for broussoside C. Figures S13–S16, the spectroscopic data of the ^1^H-NMR, ^13^C-NMR, HSQC, HMBC for broussoside D. Figures S17–S20, the spectroscopic data of the ^1^H-NMR, ^13^C-NMR, HSQC, HMBC for broussoside E.
(ZIP)

## References

[1] Suvannang N, Nantasenamat C, Isarankura-Na-Ayudhya C, Prachayasittikul V (2011) Molecular docking of aromatase inhibitors. Molecules 16: 3597–3617.

[2] Bonfield K, Amato E, Bankemper T, Agard H, Steller J, et al (2012) Development of a new class of aromatase inhibitors: Design, synthesis and inhibitory activity of 3-phenylchroman-4-one (isoflavanone) derivatives. Bioorgan Med Chem 20: 2603–2613. 10.1016/j.bmc.2012.02.042 PMC3933949 22444875

[3] Shi J, Zhang XY, Ma ZJ, Zhang M, Sun F (2010) Characterization of aromatase binding agents from the dichloromethane extract of *Corydalis yanhusuo* using ultrafiltration and liquid chromatography tandem mass spectrometry. Molecules 15: 3556–3566. 20657498 10.3390/molecules15053556 PMC6263280

[4] Yang YH, Peng K, Liu XY, Zhao DM, Duan JD, et al (2012) Effects of sex steroids on expression of myostatin in rare minnow, *Gobiocypris rarus* . Aquaculture 350: 1–7.

[5] Reyes JM, Murcia C, Zarco L, Alvarez L (2012) Progesterone concentrations in milk of CIDR-treated goats. Small Ruminant Res 106: 178–180.

[6] Cooke PS, Naaz A (2004) Role of estrogens in adipocyte development and function. Exp Biol Med 229: 1127–1135. 10.1177/153537020422901107 15564439

[7] Alu’datt MH, Rababah T, Ereifej K, Alli I (2013) Distribution, antioxidant and characterisation of phenolic compounds in soybeans, flaxseed and olives. Food Chem 139: 93–99. 23561083 10.1016/j.foodchem.2012.12.061

[8] Yang CY, Li F, Zhang XL, Wang L, Zhou ZQ, et al (2013) Phenolic antioxidants from *Rosa soulieana* flowers. Nat Prod Res 27: 2055–2058. 23805936 10.1080/14786419.2013.811660

[9] Tarola AM, Van de Velde F, Salvagni L, Preti R (2013) Determination of phenolic compounds in strawberries (*Fragaria ananassa* Duch) by high performance liquid chromatography with diode array detection. Food Anal Method 6: 227–237.

[10] Nair VD, Panneerselvam R, Gopi R, Shao HB (2013) Elicitation of pharmacologically active phenolic compounds from *Rauvolfia serpentina* Benth. Ex. Kurtz. Ind Crop Prod 45: 406–415.

[11] Bao L, Cui WF, Yu JP, Yu K, Wang MK (2010) Chemical constituents with α-glycosidase inhibiting activity from the bark of *Broussonetia papyrifera*. Nat Prod Res Dev 22: 934–936, 939.

[12] Ryu HW, Lee BW, Curtis-Long MJ, Jung S, Ryu YB, et al (2010) Polyphenols from *Broussonetia papyrifera* displaying potent alpha-glucosidase inhibition. J Agr Food Chem 58: 202–208. 19954213 10.1021/jf903068k

[13] Zheng ZP, Cheng KW, Chao JF, Wu JJ, Wang MF (2008) Tyrosinase inhibitors from paper mulberry (*Broussonetia papyrifera*). Food Chem 106: 529–535.

[14] Xu ML, Wang L, Hu JH, Lee SK, Wang MH (2010) Antioxidant activities and related polyphenolic constituents of the methanol extract fractions from *Broussonetia papyrifera* stem bark and wood. Food Sci Biotechnol 19: 677–682.

[15] Kim SY, Kim JH, Kim SK, Oh MJ, Jung MY (1994) Antioxidant activities of selected oriental herb extracts. J Am Oil Chem Soc 71: 633–640.

[16] Xiong Y, Zhao Y, Yang Y, Yang C, Ding Z, et al (2009) Extraction of the flavone from *Broussonetia papyrifera* vent roots and its anti-oxidization in vitro. China Forest Sci Technol 23: 42–45.

[17] Minglu X, Bo T, Pengfei J, Suhua G, Yan Y (2011) Research progress of chemical constituents and pharmacological activity of *Broussonetia papyrifera* at home and abroad. J Henan Institute Sci Technol 39: 51–55.

[18] Nishi Y, Yanase T, Mu YM, Oba K, Ichino I, et al (2001) Establishment and characterization of a steroidogenic human granulosa-like tumor cell line, KGN, that expresses functional follicle-stimulating hormone receptor. Endocrinology 142: 437–445. 11145608 10.1210/endo.142.1.7862

[19] Lu DF, Yang LJ, Wang F, Zhang GL (2012) Inhibitory effect of luteolin on estrogen biosynthesis in human ovarian granulosa cells by suppression of aromatase (CYP19). J Agr Food Chem 60: 8411–8418. 22838964 10.1021/jf3022817

[20] Yang C, Yang Y, Aisa HA, Xin X, Ma H, et al (2012) Bioassay-guided isolation of antioxidants from *Astragalus altaicus* by combination of chromatographic techniques. J Sep Sci 35: 977–983. 22589158 10.1002/jssc.201101104

[21] Zhu LF, Xu M, Zhu HT, Wang D, Yang SX, et al (2012) New flavan-3-ol dimer from green tea produced from *Camellia taliensis* in the Ai-Lao mountains of southwest China. J Agr Food Chem 60: 12170–12176. 23167720 10.1021/jf302726t

[22] Yang LJ, Lu DF, Guo JJ, Meng XL, Zhang GL, et al (2013) Icariin from *Epimedium brevicornum* Maxim promotes the biosynthesis of estrogen by aromatase (CYP19). J Ethnopharmacol 145: 715–721. 23261485 10.1016/j.jep.2012.11.031

[23] Stauffer F, Furet P, Floersheimer A, Lang M (2012) New aromatase inhibitors from the 3-pyridyl arylether and 1-aryl pyrrolo[2,3-c]pyridine series. Bioorg Med Chem Lett 22: 1860–1863. 22335894 10.1016/j.bmcl.2012.01.076

[24] McInnes KJ, Brown KA, Knower KC, Chand AL, Clyne CD, et al (2008) Characterisation of aromatase expression in the human adipocyte cell line SGBS. Breast Cancer Res Tr 112: 429–435. 10.1007/s10549-007-9883-2 18181018

[25] Tang WW, Xu HH, Zeng DQ, Yu LJ (2012) The antifungal constituents from the seeds of *Itoa orientalis* . Fitoterapia 83: 513–517. 22233862 10.1016/j.fitote.2011.12.016

[26] Yi B, Hu LF, Mei WL, Zhou KB, Wang H, et al (2011) Antioxidant phenolic compounds of cassava (*Manihot esculenta*) from Hainan. Molecules 16: 10157–10167. 22157579 10.3390/molecules161210157 PMC6264345

[27] Gerothanassis IP, Exarchou V, Lagouri V, Troganis A, Tsimidou M, et al (1998) Methodology for identification of phenolic acids in complex phenolic mixtures by high-resolution two-dimensional nuclear magnetic resonance. Application to methanolic extracts of two oregano species. J Agr Food Chem 46: 4185–4192.

[28] Bhattacharya P, Saha A (2013) Evaluation of reversible contraceptive potential of *Cordia dichotoma* leaves extract. Rev Bras Farmacogn 23: 342–350.

[29] Sashidhara KV, Singh SP, Singh SV, Srivastava RK, Srivastava K, et al (2013) Isolation and identification of beta-hematin inhibitors from *Flacourtia indica* as promising antiplasmodial agents. Eur J Med Chem 60: 497–502. 23354072 10.1016/j.ejmech.2012.12.019

[30] Ouyang MA, Wein YS, Zhang ZK, Kuo YH (2007) Inhibitory activity against tobacco mosaic virus (TMV) replication of pinoresinol and syringaresinol lignans and their glycosides from the root of *Rhus javanica* var. *roxburghiana* . J Agr Food Chem 55: 6460–6465. 17616139 10.1021/jf0709808

[31] Bhaumik PK, Guha KP, Biswas GK, Mukherjee B (1987) (-)Flacourtin, a phenolic glucoside ester from *Flacourtia indica* . Phytochemistry 26: 3090–3091.

[32] Kisiel W, Barszcz B (2000) Further sesquiterpenoids and phenolics from *Taraxacum officinale* . Fitoterapia 71: 269–273. 10844166 10.1016/s0367-326x(99)00158-6

[33] Lee JH, Park KH, Lee MH, Kim HT, Seo WD, et al (2013) Identification, characterisation, and quantification of phenolic compounds in the antioxidant activity-containing fraction from the seeds of Korean perilla (*Perilla frutescens*) cultivars. Food Chem 136: 843–852. 23122135 10.1016/j.foodchem.2012.08.057

[34] Shibano M, Kakutani K, Taniguchi M, Yasuda M, Baba K (2008) Antioxidant constituents in the dayflower (*Commelina communis* L.) and their alpha-glucosidase-inhibitory activity. J Nat Med 62: 349–353. 18409066 10.1007/s11418-008-0244-1

[35] Yao Y, Cheng XZ, Wang LX, Wang SH, Ren GX (2011) A determination of potential alpha-glucosidase inhibitors from azuki beans (*Vigna angularis*). Int J Mol Sci 12: 6445–6451. 22072898 10.3390/ijms12106445 PMC3210989

[36] Mun’im A, Negishi O, Ozawa T (2003) Antioxidative compounds from *Crotalaria sessiliflora* . Biosci Biotech Bioch 67: 410–414. 10.1271/bbb.67.410 12729010

[37] Shoeb M, Jaspars M, MacManns SM, Celik S, Nahar L, et al (2007) Anti-colon cancer potential of phenolic compounds from the aerial parts of *Centaurea gigantea* (Asteraceae). J Nat Med 61: 164–169.

[38] Krafczyk N, Glomb MA (2008) Characterization of phenolic compounds in rooibos tea. J Agr Food Chem 56: 3368–3376. 18399640 10.1021/jf703701n

[39] Ter Veld MGR, Schouten B, Louisse J, van Es DS, van der Saag PT, et al (2006) Estrogenic potency of food-packaging-associated plasticizers and antioxidants as detected in ER alpha and ER beta reporter gene cell lines. J Agr Food Chem 54: 4407–4416. 16756374 10.1021/jf052864f

[40] Lee D, Bhat KPL, Fong HHS, Farnsworth NR, Pezzuto JM, et al (2001) Aromatase inhibitors from *Broussonetia papyrifera* . J Nat Prod 64: 1286–1293. 11678652 10.1021/np010288l

[41] Gao DF, Xu M, Zhao P, Zhang XY, Wang YF, et al (2011) Kaempferol acetylated glycosides from the seed cake of *Camellia oleifera* . Food Chem 124: 432–436.

[42] Gollapudi SR, Telikepalli H, Keshavarzshokri A, Vandervelde D, Mitscher LA (1989) Glepidotin C: a minor antimicrobial bibenzyl from *Glycyrrhiza lepidota* . Phytochemistry 28: 3556–3557.

[43] Du QZ, Li B (2012) Identification of antioxidant compounds of *Mucuna sempervirens* by high-speed counter-current chromatographic separation-DPPH radical scavenging detection and their oestrogenic activity. Food Chem 131: 1181–1186.

[44] Pineda B, Garcia-Perez MA, Cano A, Lluch A, Eroles P (2013) Associations between aromatase CYP19 rs10046 polymorphism and breast anccer risk: From a case-control to a meta-analysis of 20,098 subjects. Plos One 8: 1–9. 10.1371/journal.pone.0053902 PMC3547044 23342035

[45] Dawidowicz AL, Olszowy M (2013) The importance of solvent type in estimating antioxidant properties of phenolic compounds by ABTS assay. Eur Food Res Technol 236: 1099–1105.

[46] Sarkar A, Middya TR, Jana AD (2012) A QSAR study of radical scavenging antioxidant activity of a series of flavonoids using DFT based quantum chemical descriptors - the importance of group frontier electron density. J Mol Model 18: 2621–2631. 22080306 10.1007/s00894-011-1274-2

[47] Liu YB, Singh D, Nair MG (2012) Pods of Khejri (*Prosopis cineraria*) consumed as a vegetable showed functional food properties. J Funct Foods 4: 116–121.

