# Supplementary figures and images for: Isolation and Characterization of New Phenolic Compounds with Estrogen Biosynthesis-Inhibiting and Antioxidation Activities from Broussonetia papyrifera Leaves
Source: PLoS One. 2014 Apr 8;9(4):e94198. doi: 10.1371/journal.pone.0094198 (PMC3979757; doi:10.1371/journal.pone.0094198)

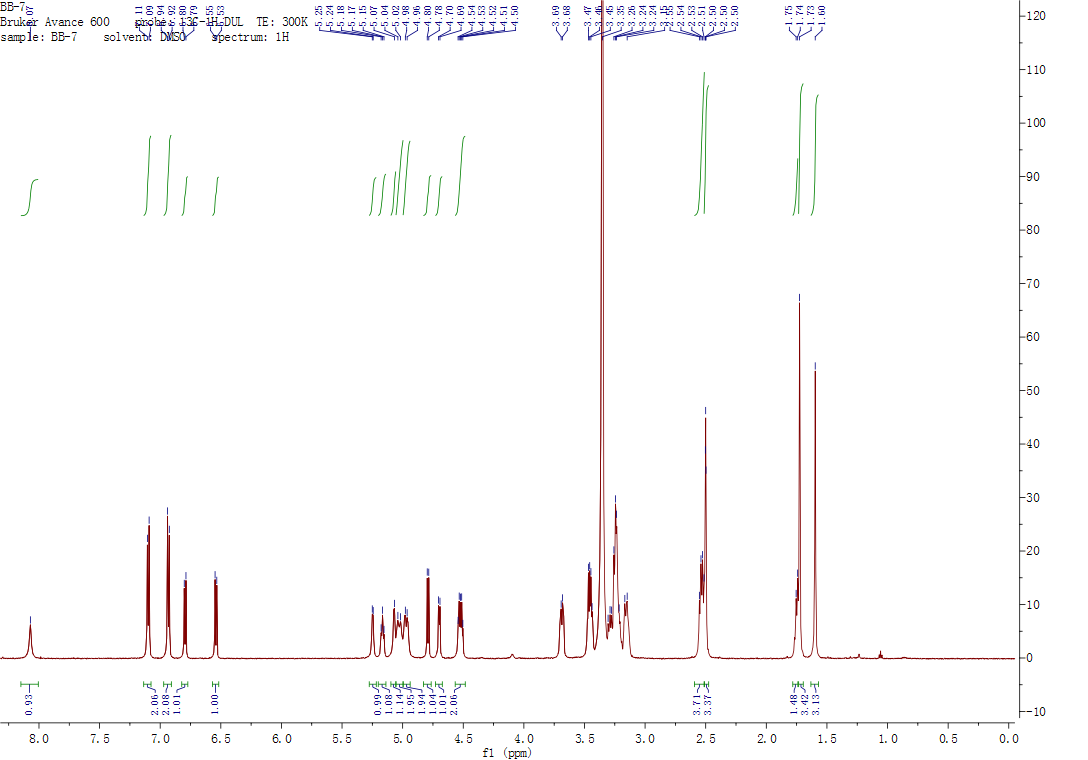

Supplement: File S1 — Figures S1–S4, the spectroscopic data of the 1H-NMR, 13C-NMR, HSQC, HMBC for broussoside A. Figures S5–S8, the spectroscopic data of the 1H-NMR, 13C-NMR, HSQC, HMBC for broussoside B. Figures S9–S12, the spectroscopic data of the 1H-NMR, 13C-NMR, HSQC, HMBC for broussoside C. Figures S13–S16, the spectroscopic data of the 1H-NMR, 13C-NMR, HSQC, HMBC for broussoside D. Figures S17–S20, the spectroscopic data of the 1H-NMR, 13C-NMR, HSQC, HMBC for broussoside E. (ZIP) [file pone.0094198.s001.zip › Figures S1-20/Figure S1 1H-NMR of broussoside A.tiff]

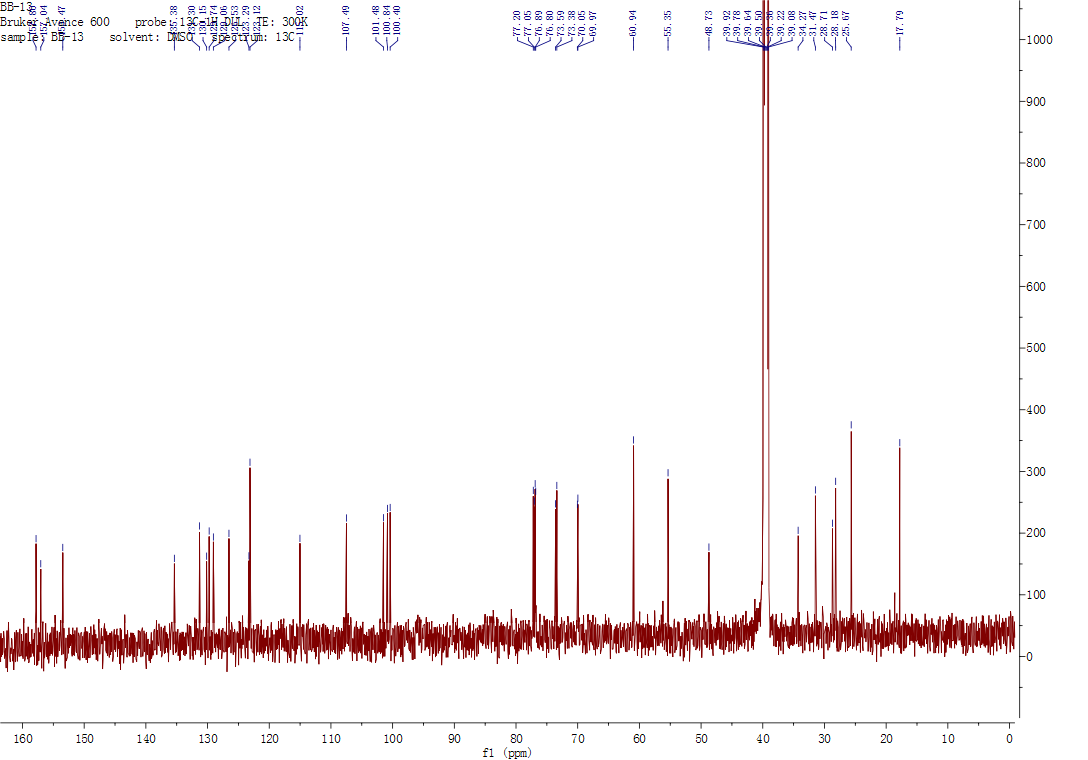

Supplement: File S1 — Figures S1–S4, the spectroscopic data of the 1H-NMR, 13C-NMR, HSQC, HMBC for broussoside A. Figures S5–S8, the spectroscopic data of the 1H-NMR, 13C-NMR, HSQC, HMBC for broussoside B. Figures S9–S12, the spectroscopic data of the 1H-NMR, 13C-NMR, HSQC, HMBC for broussoside C. Figures S13–S16, the spectroscopic data of the 1H-NMR, 13C-NMR, HSQC, HMBC for broussoside D. Figures S17–S20, the spectroscopic data of the 1H-NMR, 13C-NMR, HSQC, HMBC for broussoside E. (ZIP) [file pone.0094198.s001.zip › Figures S1-20/Figure S10 13C-NMR of broussoside C.tiff]

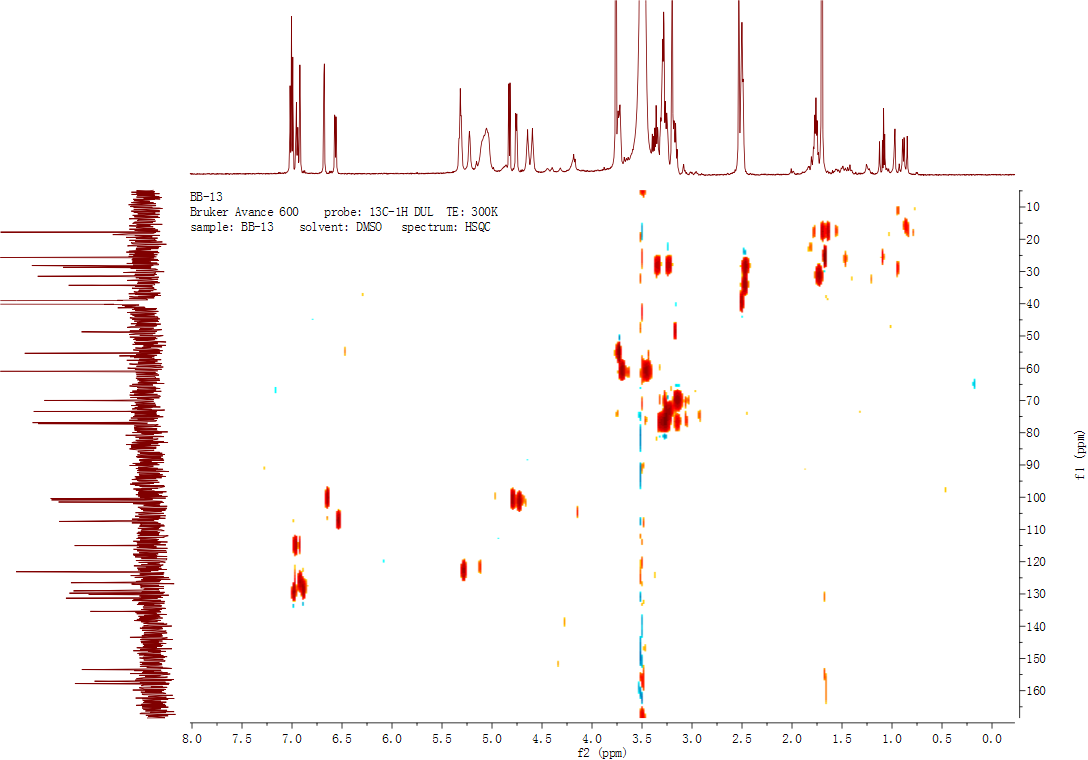

Supplement: File S1 — Figures S1–S4, the spectroscopic data of the 1H-NMR, 13C-NMR, HSQC, HMBC for broussoside A. Figures S5–S8, the spectroscopic data of the 1H-NMR, 13C-NMR, HSQC, HMBC for broussoside B. Figures S9–S12, the spectroscopic data of the 1H-NMR, 13C-NMR, HSQC, HMBC for broussoside C. Figures S13–S16, the spectroscopic data of the 1H-NMR, 13C-NMR, HSQC, HMBC for broussoside D. Figures S17–S20, the spectroscopic data of the 1H-NMR, 13C-NMR, HSQC, HMBC for broussoside E. (ZIP) [file pone.0094198.s001.zip › Figures S1-20/Figure S11 HSQC of broussoside C.tiff]

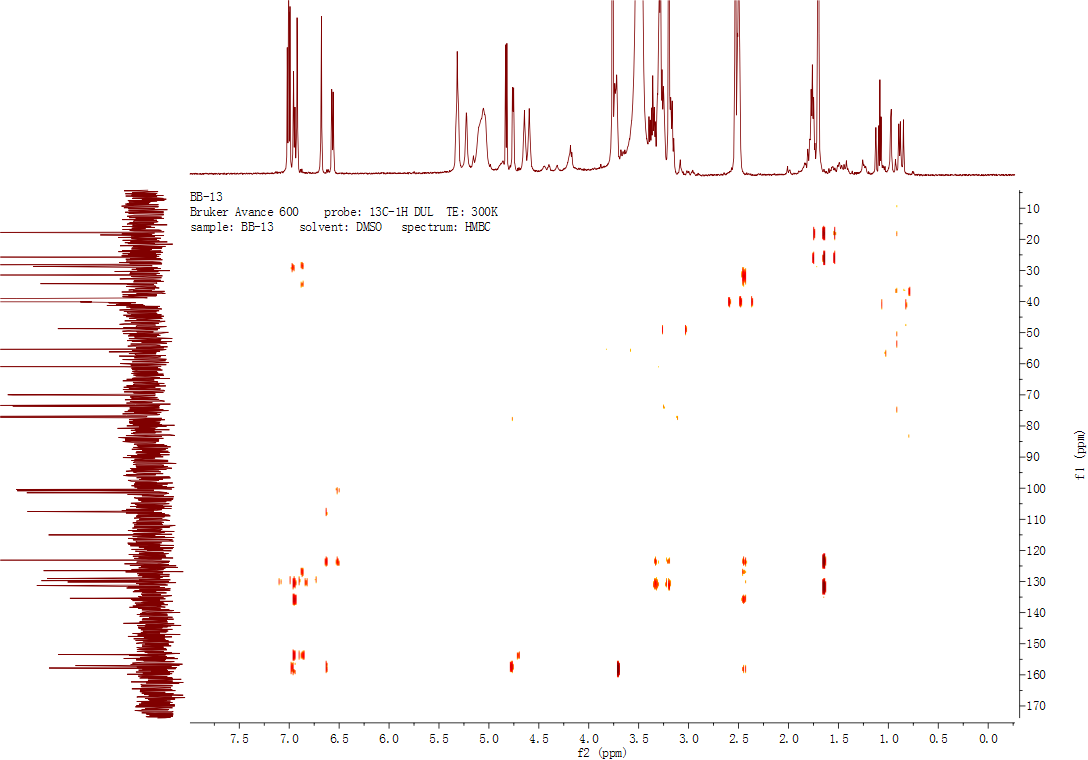

Supplement: File S1 — Figures S1–S4, the spectroscopic data of the 1H-NMR, 13C-NMR, HSQC, HMBC for broussoside A. Figures S5–S8, the spectroscopic data of the 1H-NMR, 13C-NMR, HSQC, HMBC for broussoside B. Figures S9–S12, the spectroscopic data of the 1H-NMR, 13C-NMR, HSQC, HMBC for broussoside C. Figures S13–S16, the spectroscopic data of the 1H-NMR, 13C-NMR, HSQC, HMBC for broussoside D. Figures S17–S20, the spectroscopic data of the 1H-NMR, 13C-NMR, HSQC, HMBC for broussoside E. (ZIP) [file pone.0094198.s001.zip › Figures S1-20/Figure S12 HMBC of broussoside C.tiff]

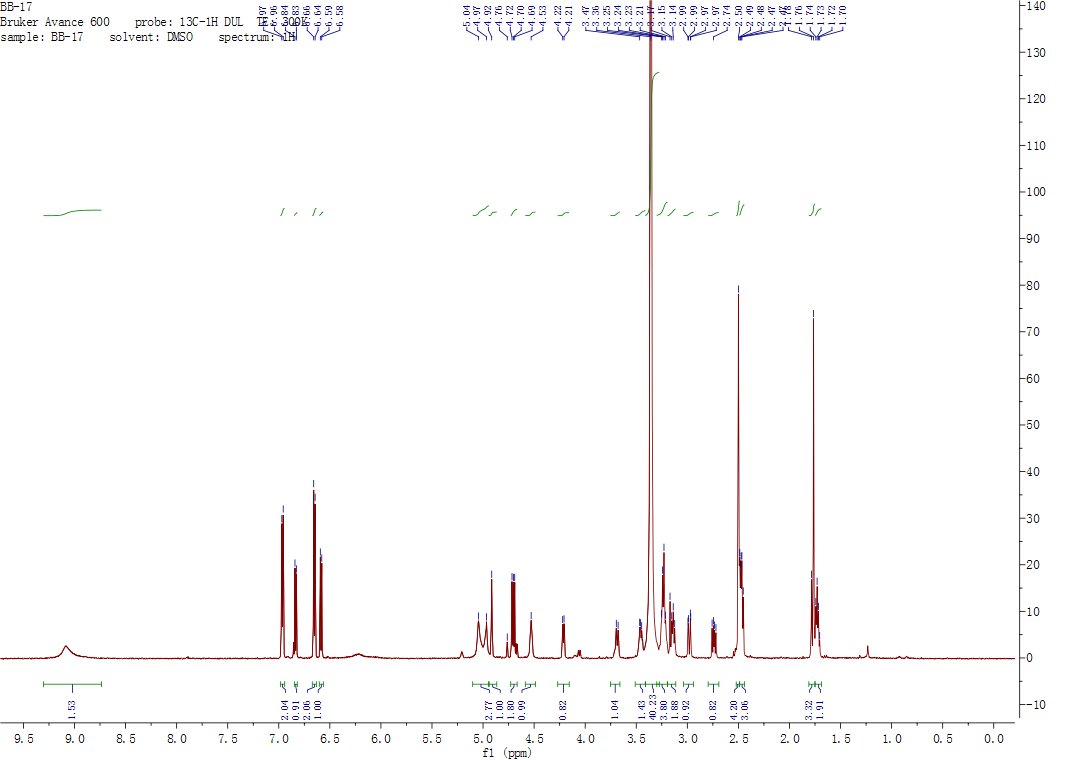

Supplement: File S1 — Figures S1–S4, the spectroscopic data of the 1H-NMR, 13C-NMR, HSQC, HMBC for broussoside A. Figures S5–S8, the spectroscopic data of the 1H-NMR, 13C-NMR, HSQC, HMBC for broussoside B. Figures S9–S12, the spectroscopic data of the 1H-NMR, 13C-NMR, HSQC, HMBC for broussoside C. Figures S13–S16, the spectroscopic data of the 1H-NMR, 13C-NMR, HSQC, HMBC for broussoside D. Figures S17–S20, the spectroscopic data of the 1H-NMR, 13C-NMR, HSQC, HMBC for broussoside E. (ZIP) [file pone.0094198.s001.zip › Figures S1-20/Figure S13 1H-NMR of broussoside D.tiff]

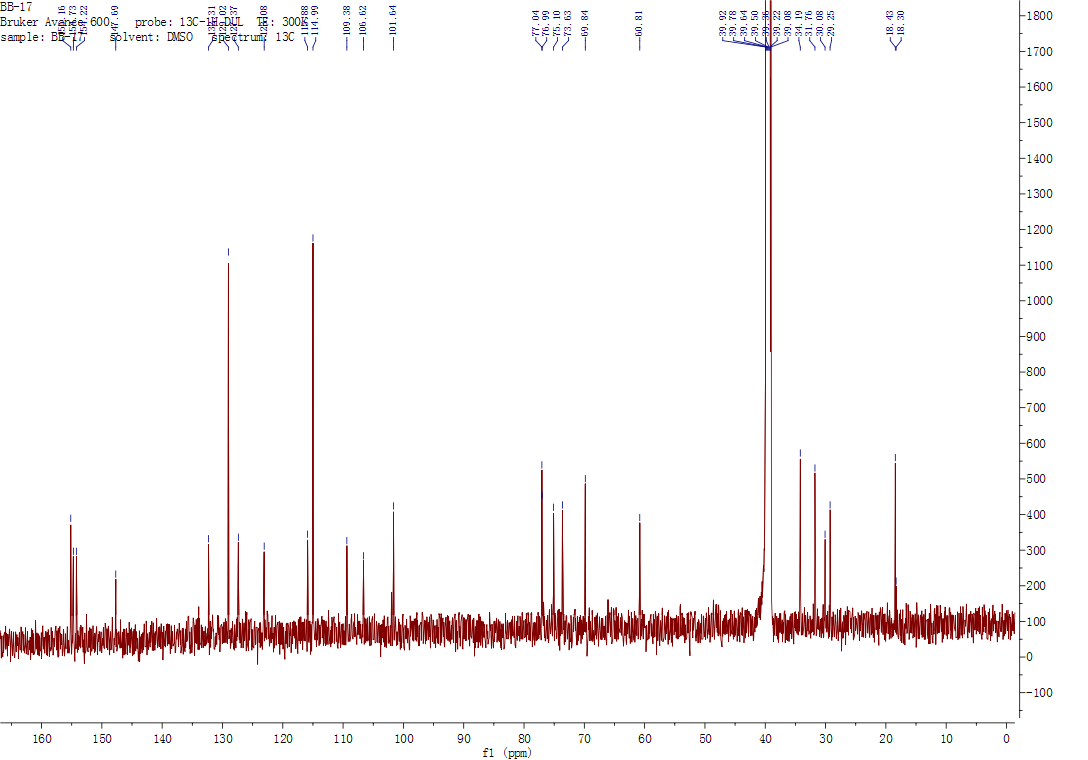

Supplement: File S1 — Figures S1–S4, the spectroscopic data of the 1H-NMR, 13C-NMR, HSQC, HMBC for broussoside A. Figures S5–S8, the spectroscopic data of the 1H-NMR, 13C-NMR, HSQC, HMBC for broussoside B. Figures S9–S12, the spectroscopic data of the 1H-NMR, 13C-NMR, HSQC, HMBC for broussoside C. Figures S13–S16, the spectroscopic data of the 1H-NMR, 13C-NMR, HSQC, HMBC for broussoside D. Figures S17–S20, the spectroscopic data of the 1H-NMR, 13C-NMR, HSQC, HMBC for broussoside E. (ZIP) [file pone.0094198.s001.zip › Figures S1-20/Figure S14 13C-NMR of broussoside D.tiff]

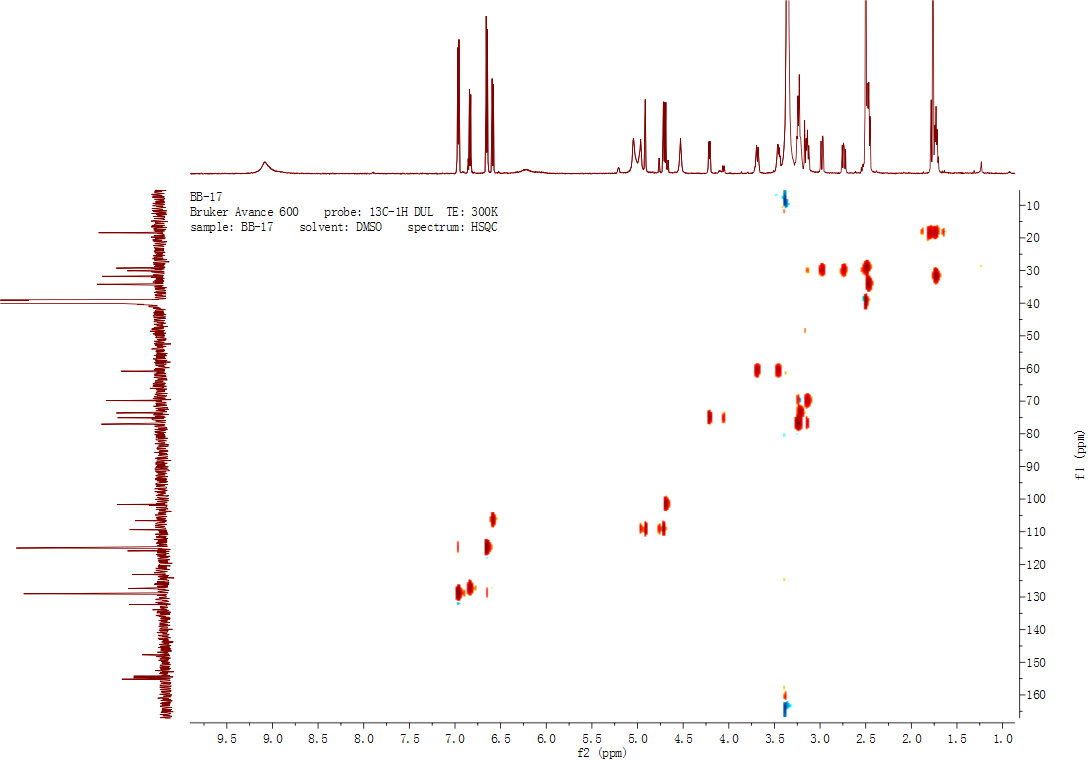

Supplement: File S1 — Figures S1–S4, the spectroscopic data of the 1H-NMR, 13C-NMR, HSQC, HMBC for broussoside A. Figures S5–S8, the spectroscopic data of the 1H-NMR, 13C-NMR, HSQC, HMBC for broussoside B. Figures S9–S12, the spectroscopic data of the 1H-NMR, 13C-NMR, HSQC, HMBC for broussoside C. Figures S13–S16, the spectroscopic data of the 1H-NMR, 13C-NMR, HSQC, HMBC for broussoside D. Figures S17–S20, the spectroscopic data of the 1H-NMR, 13C-NMR, HSQC, HMBC for broussoside E. (ZIP) [file pone.0094198.s001.zip › Figures S1-20/Figure S15 HSQC of broussoside D.tiff]

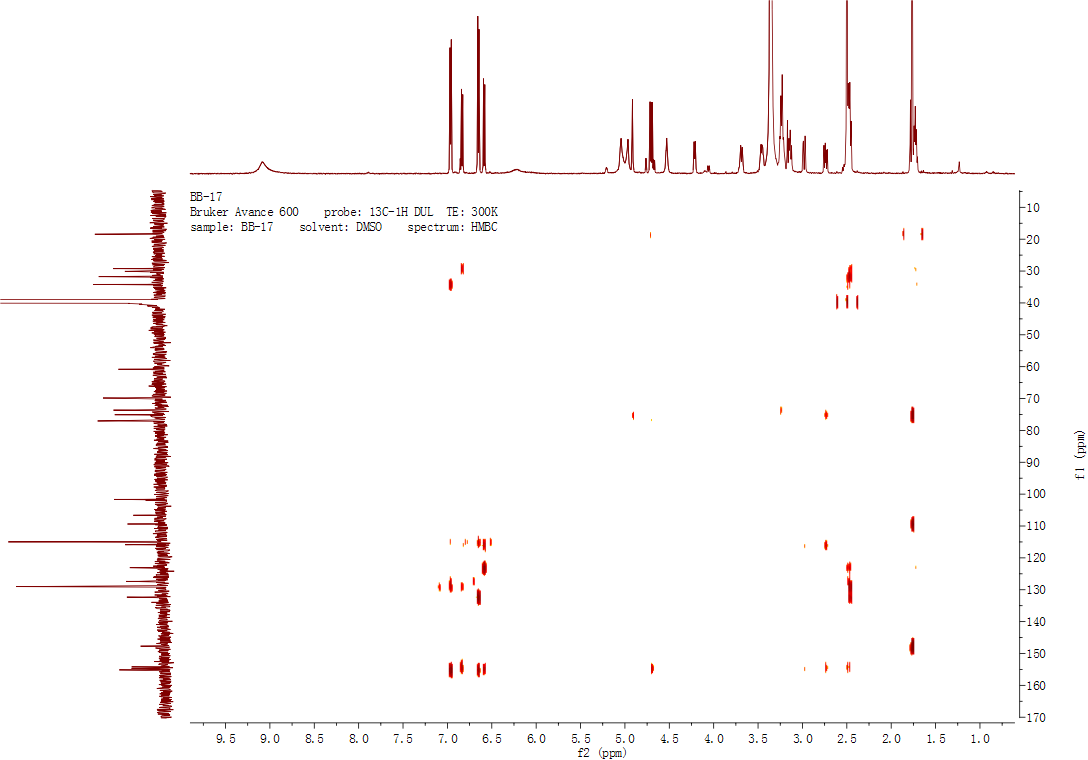

Supplement: File S1 — Figures S1–S4, the spectroscopic data of the 1H-NMR, 13C-NMR, HSQC, HMBC for broussoside A. Figures S5–S8, the spectroscopic data of the 1H-NMR, 13C-NMR, HSQC, HMBC for broussoside B. Figures S9–S12, the spectroscopic data of the 1H-NMR, 13C-NMR, HSQC, HMBC for broussoside C. Figures S13–S16, the spectroscopic data of the 1H-NMR, 13C-NMR, HSQC, HMBC for broussoside D. Figures S17–S20, the spectroscopic data of the 1H-NMR, 13C-NMR, HSQC, HMBC for broussoside E. (ZIP) [file pone.0094198.s001.zip › Figures S1-20/Figure S16 HMBC of broussoside D.tiff]

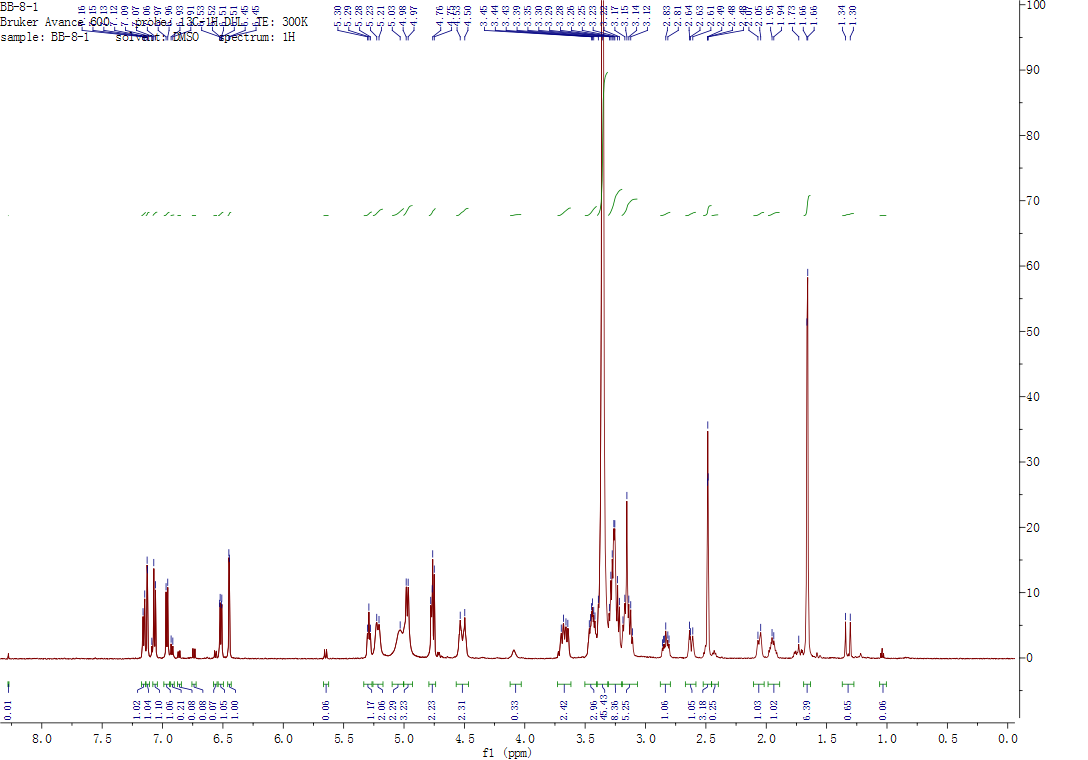

Supplement: File S1 — Figures S1–S4, the spectroscopic data of the 1H-NMR, 13C-NMR, HSQC, HMBC for broussoside A. Figures S5–S8, the spectroscopic data of the 1H-NMR, 13C-NMR, HSQC, HMBC for broussoside B. Figures S9–S12, the spectroscopic data of the 1H-NMR, 13C-NMR, HSQC, HMBC for broussoside C. Figures S13–S16, the spectroscopic data of the 1H-NMR, 13C-NMR, HSQC, HMBC for broussoside D. Figures S17–S20, the spectroscopic data of the 1H-NMR, 13C-NMR, HSQC, HMBC for broussoside E. (ZIP) [file pone.0094198.s001.zip › Figures S1-20/Figure S17 1H-NMR of broussoside E.tiff]

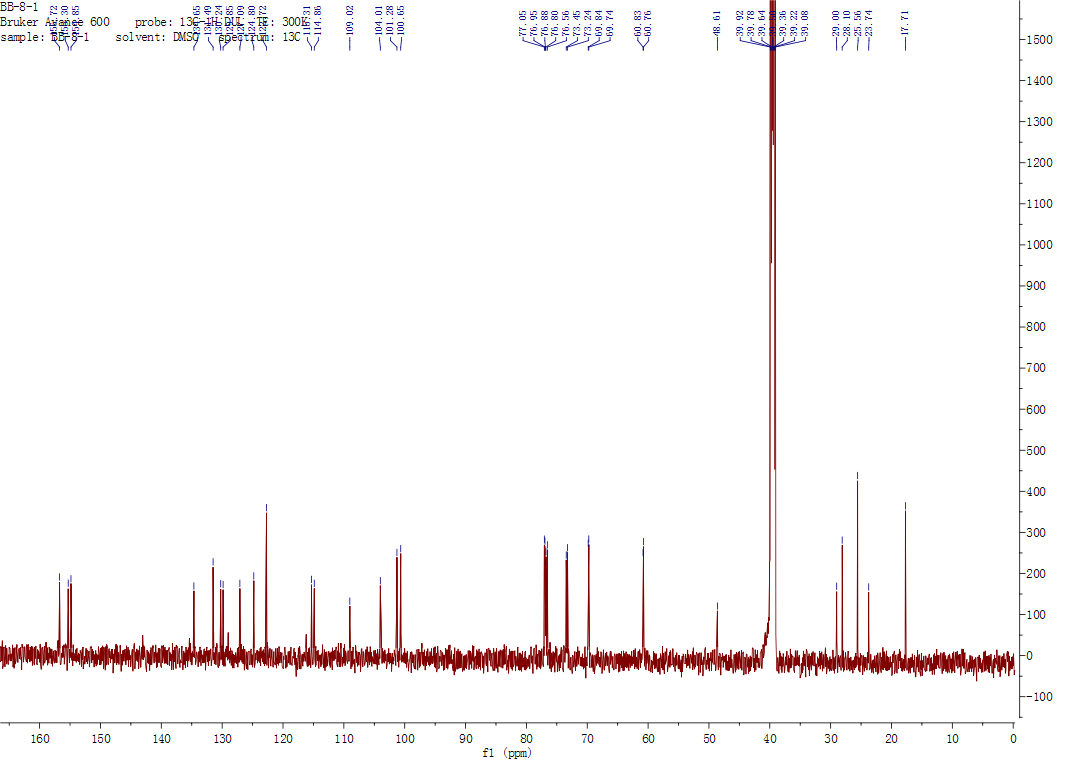

Supplement: File S1 — Figures S1–S4, the spectroscopic data of the 1H-NMR, 13C-NMR, HSQC, HMBC for broussoside A. Figures S5–S8, the spectroscopic data of the 1H-NMR, 13C-NMR, HSQC, HMBC for broussoside B. Figures S9–S12, the spectroscopic data of the 1H-NMR, 13C-NMR, HSQC, HMBC for broussoside C. Figures S13–S16, the spectroscopic data of the 1H-NMR, 13C-NMR, HSQC, HMBC for broussoside D. Figures S17–S20, the spectroscopic data of the 1H-NMR, 13C-NMR, HSQC, HMBC for broussoside E. (ZIP) [file pone.0094198.s001.zip › Figures S1-20/Figure S18 13C-NMR of broussoside E.tiff]

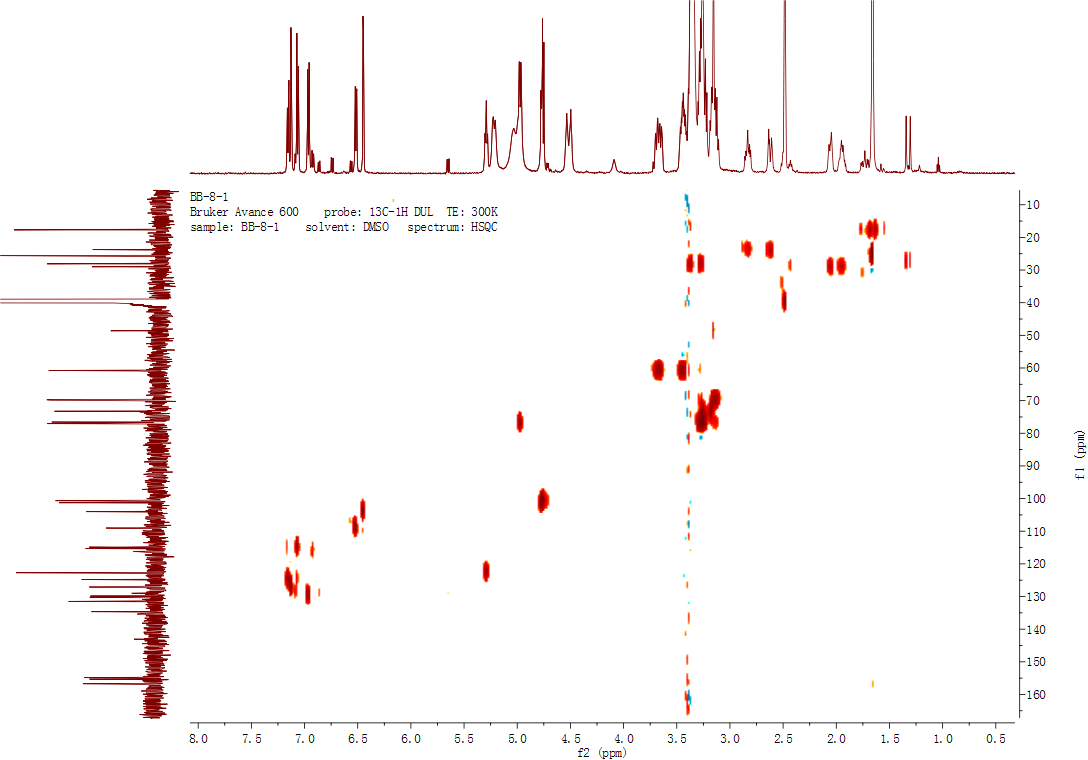

Supplement: File S1 — Figures S1–S4, the spectroscopic data of the 1H-NMR, 13C-NMR, HSQC, HMBC for broussoside A. Figures S5–S8, the spectroscopic data of the 1H-NMR, 13C-NMR, HSQC, HMBC for broussoside B. Figures S9–S12, the spectroscopic data of the 1H-NMR, 13C-NMR, HSQC, HMBC for broussoside C. Figures S13–S16, the spectroscopic data of the 1H-NMR, 13C-NMR, HSQC, HMBC for broussoside D. Figures S17–S20, the spectroscopic data of the 1H-NMR, 13C-NMR, HSQC, HMBC for broussoside E. (ZIP) [file pone.0094198.s001.zip › Figures S1-20/Figure S19 HSQC of broussoside E.tiff]

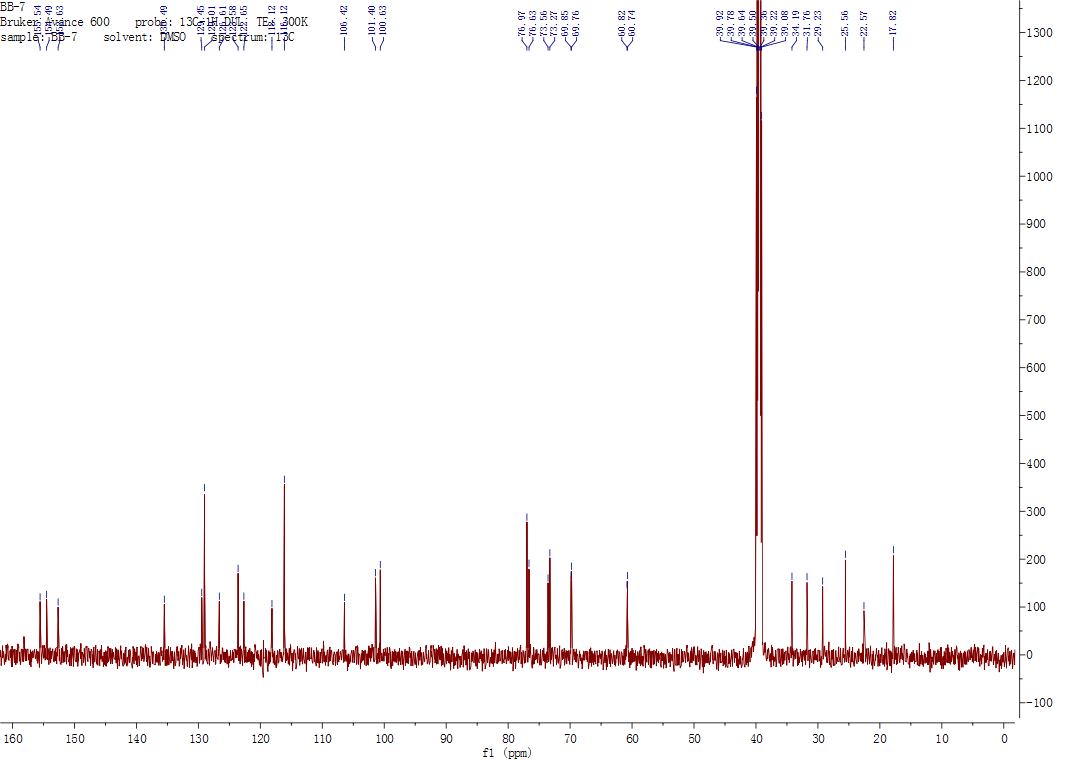

Supplement: File S1 — Figures S1–S4, the spectroscopic data of the 1H-NMR, 13C-NMR, HSQC, HMBC for broussoside A. Figures S5–S8, the spectroscopic data of the 1H-NMR, 13C-NMR, HSQC, HMBC for broussoside B. Figures S9–S12, the spectroscopic data of the 1H-NMR, 13C-NMR, HSQC, HMBC for broussoside C. Figures S13–S16, the spectroscopic data of the 1H-NMR, 13C-NMR, HSQC, HMBC for broussoside D. Figures S17–S20, the spectroscopic data of the 1H-NMR, 13C-NMR, HSQC, HMBC for broussoside E. (ZIP) [file pone.0094198.s001.zip › Figures S1-20/Figure S2 13C-NMR of broussoside A.tiff]

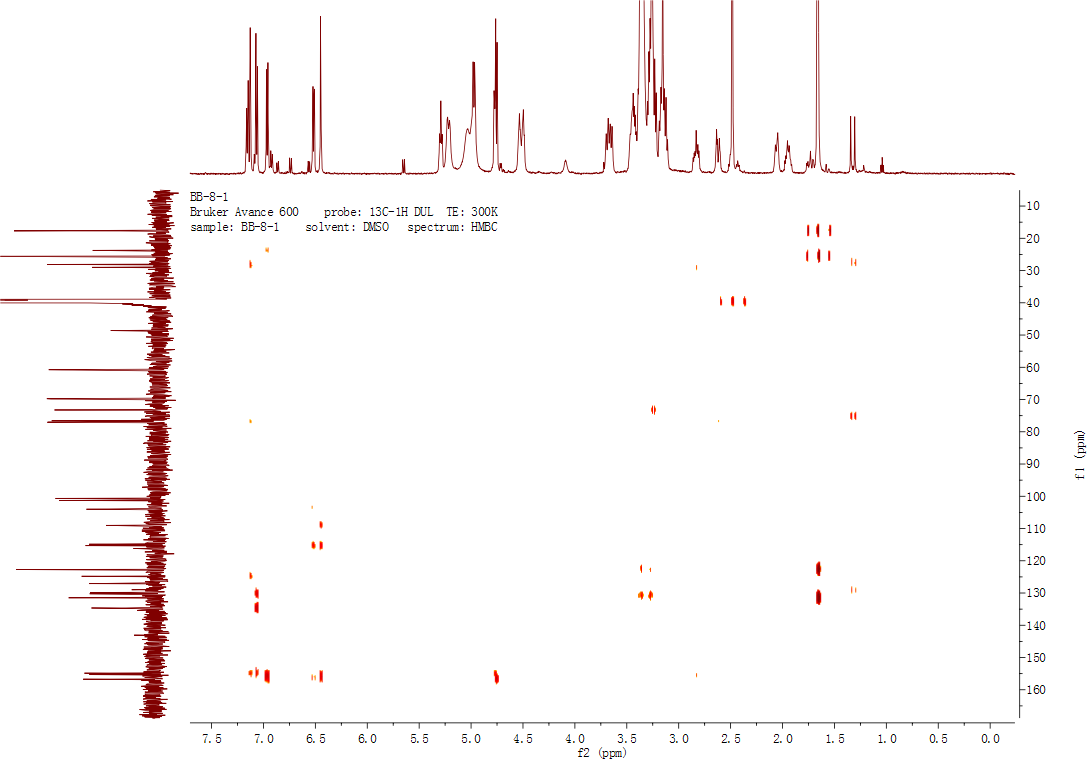

Supplement: File S1 — Figures S1–S4, the spectroscopic data of the 1H-NMR, 13C-NMR, HSQC, HMBC for broussoside A. Figures S5–S8, the spectroscopic data of the 1H-NMR, 13C-NMR, HSQC, HMBC for broussoside B. Figures S9–S12, the spectroscopic data of the 1H-NMR, 13C-NMR, HSQC, HMBC for broussoside C. Figures S13–S16, the spectroscopic data of the 1H-NMR, 13C-NMR, HSQC, HMBC for broussoside D. Figures S17–S20, the spectroscopic data of the 1H-NMR, 13C-NMR, HSQC, HMBC for broussoside E. (ZIP) [file pone.0094198.s001.zip › Figures S1-20/Figure S20 HMBC of broussoside E.tiff]

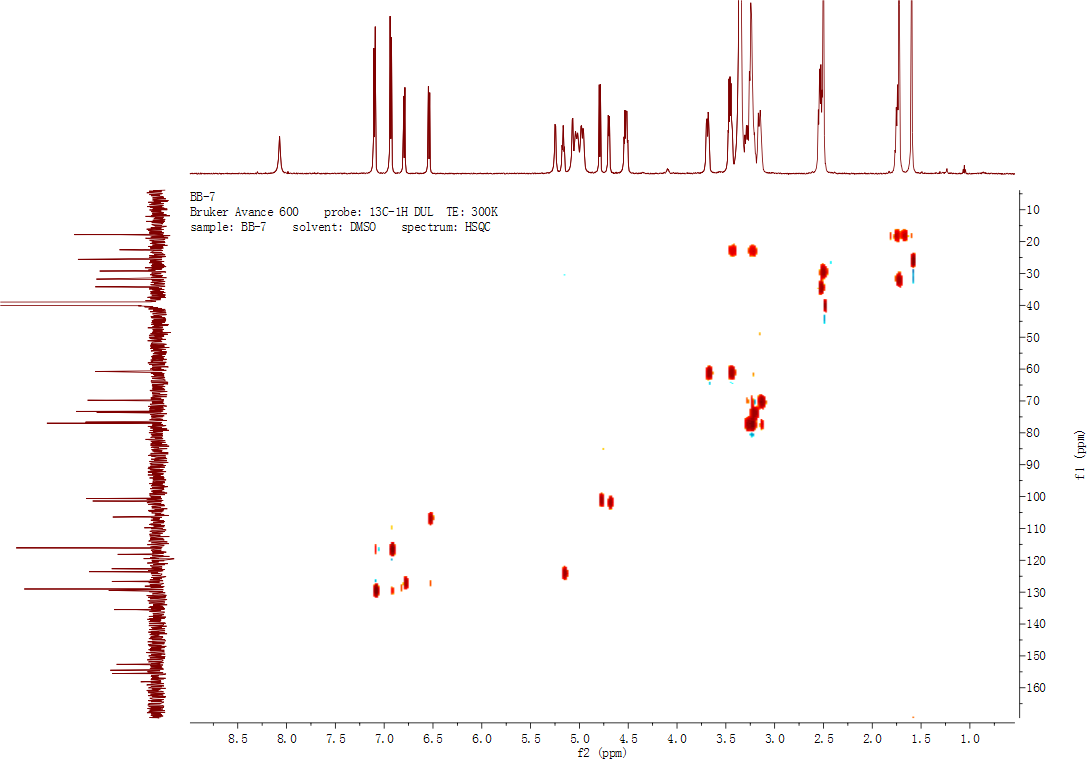

Supplement: File S1 — Figures S1–S4, the spectroscopic data of the 1H-NMR, 13C-NMR, HSQC, HMBC for broussoside A. Figures S5–S8, the spectroscopic data of the 1H-NMR, 13C-NMR, HSQC, HMBC for broussoside B. Figures S9–S12, the spectroscopic data of the 1H-NMR, 13C-NMR, HSQC, HMBC for broussoside C. Figures S13–S16, the spectroscopic data of the 1H-NMR, 13C-NMR, HSQC, HMBC for broussoside D. Figures S17–S20, the spectroscopic data of the 1H-NMR, 13C-NMR, HSQC, HMBC for broussoside E. (ZIP) [file pone.0094198.s001.zip › Figures S1-20/Figure S3 HSQC of broussoside A.tiff]

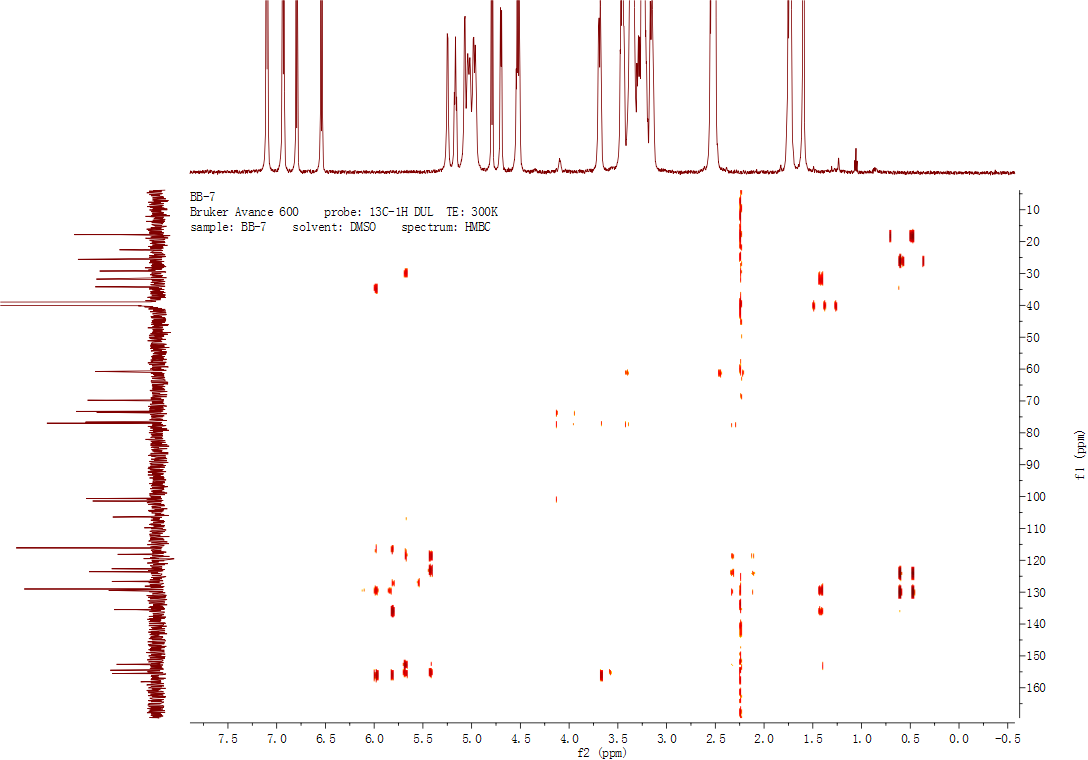

Supplement: File S1 — Figures S1–S4, the spectroscopic data of the 1H-NMR, 13C-NMR, HSQC, HMBC for broussoside A. Figures S5–S8, the spectroscopic data of the 1H-NMR, 13C-NMR, HSQC, HMBC for broussoside B. Figures S9–S12, the spectroscopic data of the 1H-NMR, 13C-NMR, HSQC, HMBC for broussoside C. Figures S13–S16, the spectroscopic data of the 1H-NMR, 13C-NMR, HSQC, HMBC for broussoside D. Figures S17–S20, the spectroscopic data of the 1H-NMR, 13C-NMR, HSQC, HMBC for broussoside E. (ZIP) [file pone.0094198.s001.zip › Figures S1-20/Figure S4 HMBC of broussoside A.tiff]

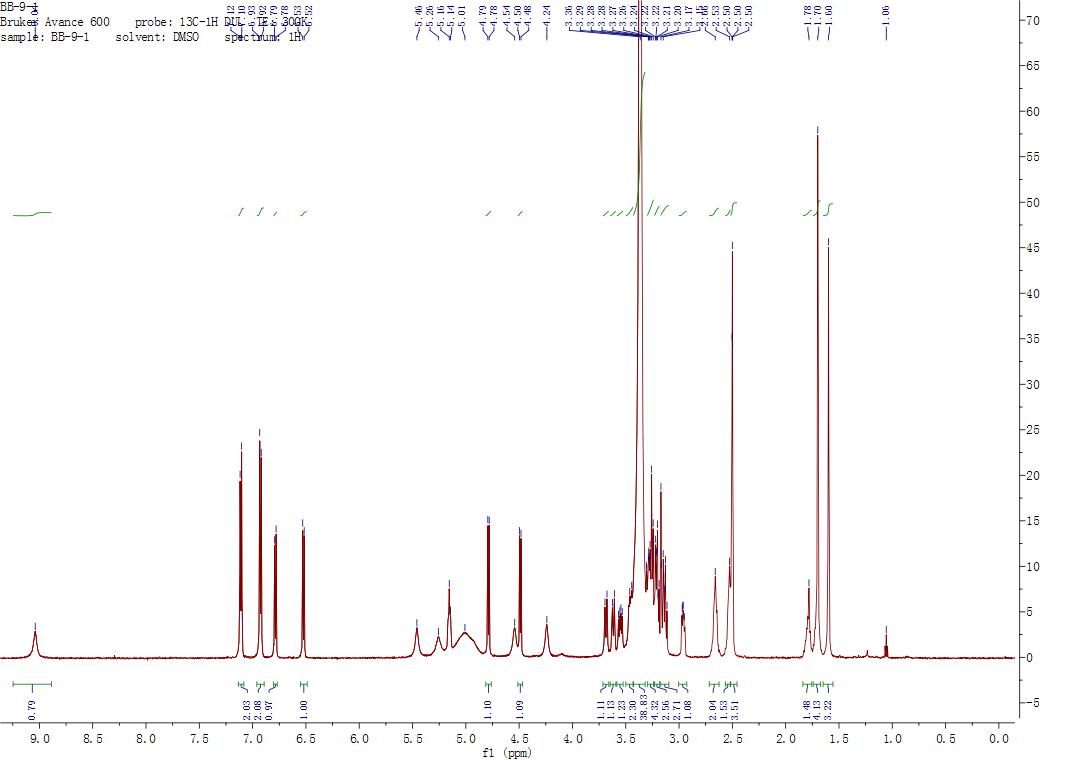

Supplement: File S1 — Figures S1–S4, the spectroscopic data of the 1H-NMR, 13C-NMR, HSQC, HMBC for broussoside A. Figures S5–S8, the spectroscopic data of the 1H-NMR, 13C-NMR, HSQC, HMBC for broussoside B. Figures S9–S12, the spectroscopic data of the 1H-NMR, 13C-NMR, HSQC, HMBC for broussoside C. Figures S13–S16, the spectroscopic data of the 1H-NMR, 13C-NMR, HSQC, HMBC for broussoside D. Figures S17–S20, the spectroscopic data of the 1H-NMR, 13C-NMR, HSQC, HMBC for broussoside E. (ZIP) [file pone.0094198.s001.zip › Figures S1-20/Figure S5 1H-NMR of broussoside B.tiff]

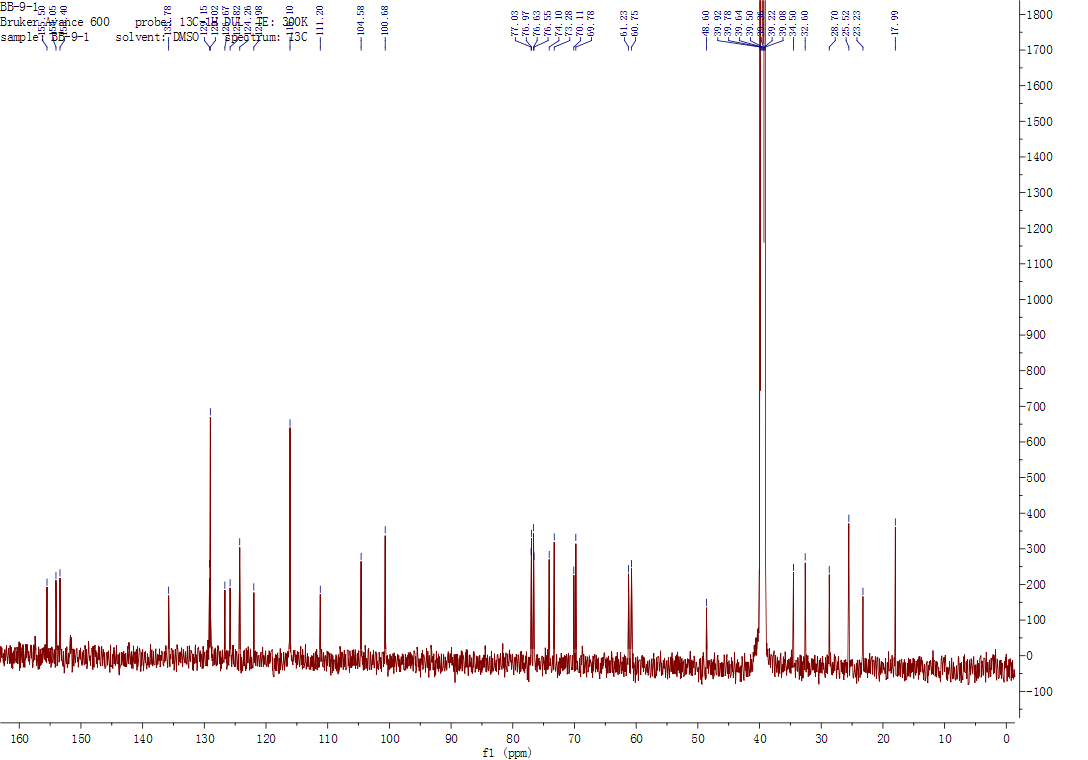

Supplement: File S1 — Figures S1–S4, the spectroscopic data of the 1H-NMR, 13C-NMR, HSQC, HMBC for broussoside A. Figures S5–S8, the spectroscopic data of the 1H-NMR, 13C-NMR, HSQC, HMBC for broussoside B. Figures S9–S12, the spectroscopic data of the 1H-NMR, 13C-NMR, HSQC, HMBC for broussoside C. Figures S13–S16, the spectroscopic data of the 1H-NMR, 13C-NMR, HSQC, HMBC for broussoside D. Figures S17–S20, the spectroscopic data of the 1H-NMR, 13C-NMR, HSQC, HMBC for broussoside E. (ZIP) [file pone.0094198.s001.zip › Figures S1-20/Figure S6 13C-NMR of broussoside B.tiff]

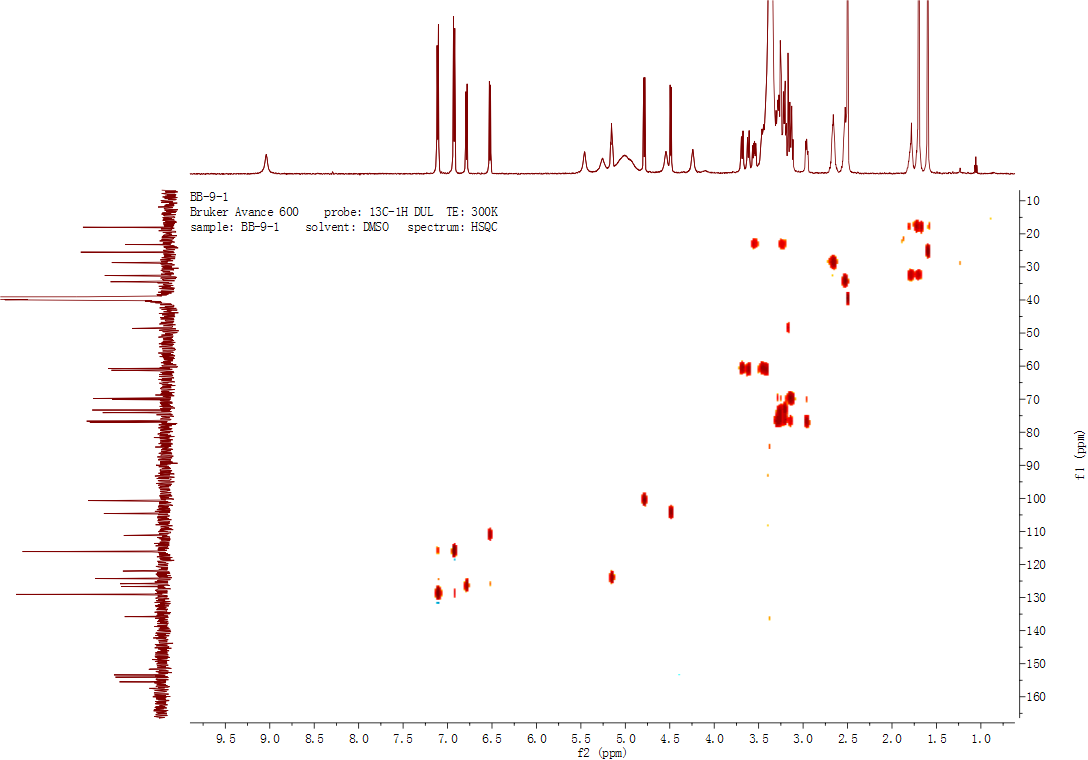

Supplement: File S1 — Figures S1–S4, the spectroscopic data of the 1H-NMR, 13C-NMR, HSQC, HMBC for broussoside A. Figures S5–S8, the spectroscopic data of the 1H-NMR, 13C-NMR, HSQC, HMBC for broussoside B. Figures S9–S12, the spectroscopic data of the 1H-NMR, 13C-NMR, HSQC, HMBC for broussoside C. Figures S13–S16, the spectroscopic data of the 1H-NMR, 13C-NMR, HSQC, HMBC for broussoside D. Figures S17–S20, the spectroscopic data of the 1H-NMR, 13C-NMR, HSQC, HMBC for broussoside E. (ZIP) [file pone.0094198.s001.zip › Figures S1-20/Figure S7 HSQC of broussoside B.tiff]

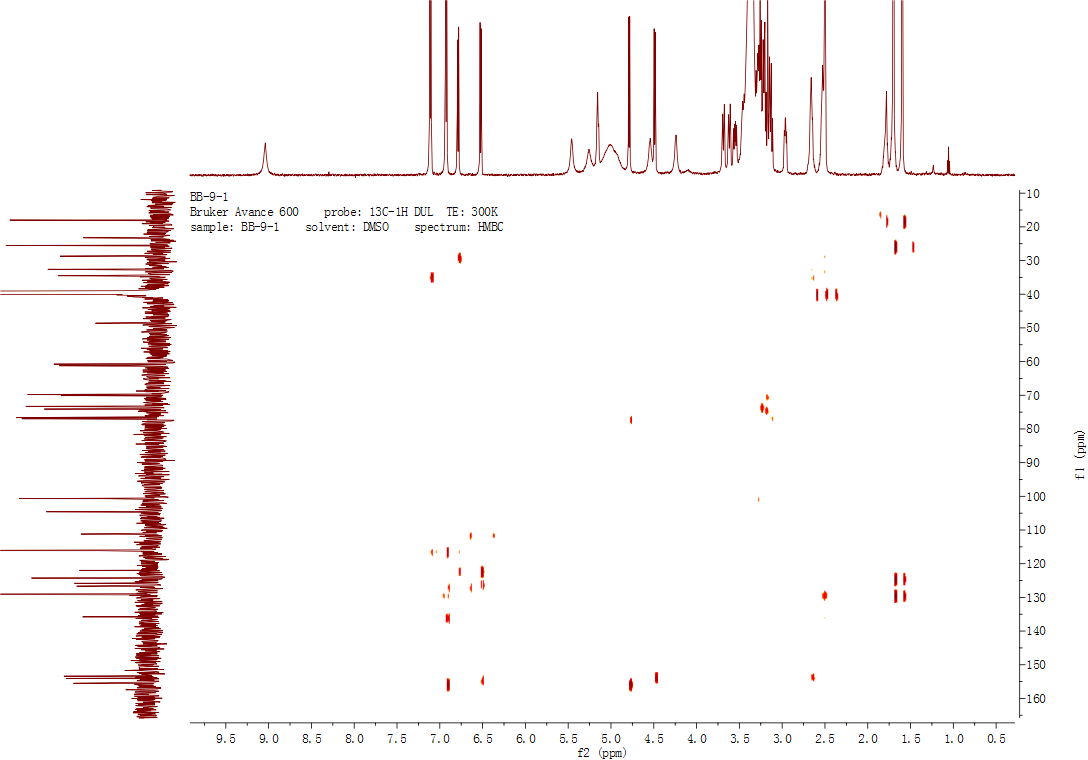

Supplement: File S1 — Figures S1–S4, the spectroscopic data of the 1H-NMR, 13C-NMR, HSQC, HMBC for broussoside A. Figures S5–S8, the spectroscopic data of the 1H-NMR, 13C-NMR, HSQC, HMBC for broussoside B. Figures S9–S12, the spectroscopic data of the 1H-NMR, 13C-NMR, HSQC, HMBC for broussoside C. Figures S13–S16, the spectroscopic data of the 1H-NMR, 13C-NMR, HSQC, HMBC for broussoside D. Figures S17–S20, the spectroscopic data of the 1H-NMR, 13C-NMR, HSQC, HMBC for broussoside E. (ZIP) [file pone.0094198.s001.zip › Figures S1-20/Figure S8 HMBC of broussoside B.tiff]

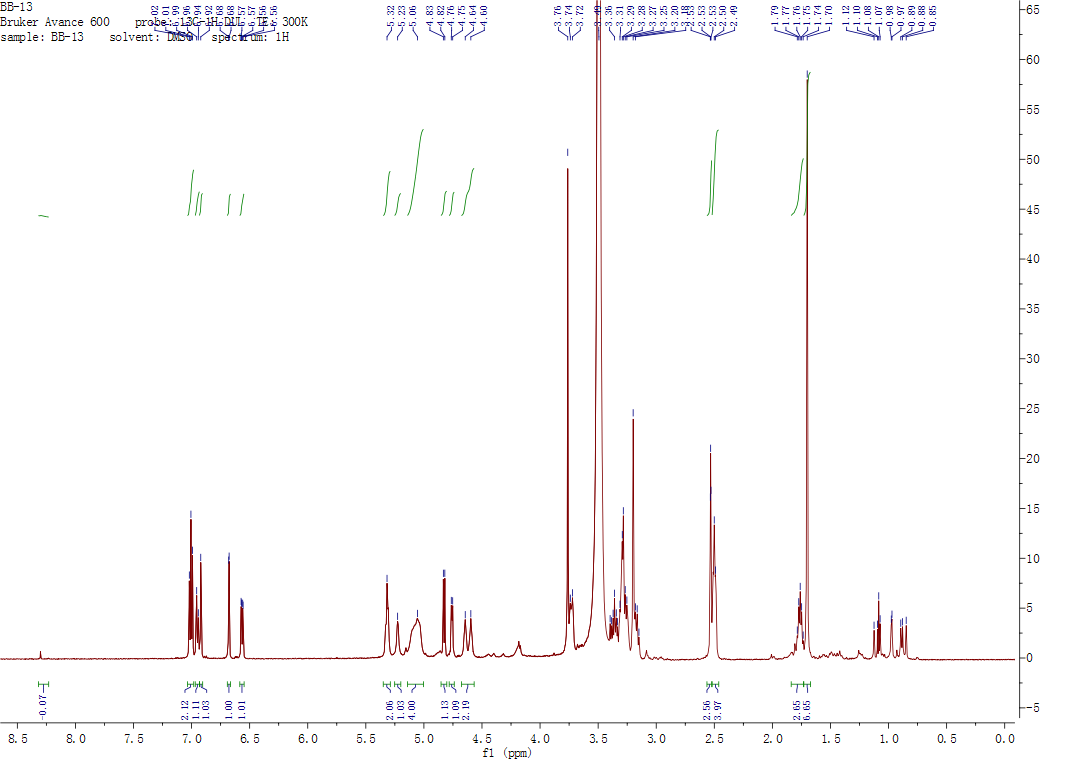

Supplement: File S1 — Figures S1–S4, the spectroscopic data of the 1H-NMR, 13C-NMR, HSQC, HMBC for broussoside A. Figures S5–S8, the spectroscopic data of the 1H-NMR, 13C-NMR, HSQC, HMBC for broussoside B. Figures S9–S12, the spectroscopic data of the 1H-NMR, 13C-NMR, HSQC, HMBC for broussoside C. Figures S13–S16, the spectroscopic data of the 1H-NMR, 13C-NMR, HSQC, HMBC for broussoside D. Figures S17–S20, the spectroscopic data of the 1H-NMR, 13C-NMR, HSQC, HMBC for broussoside E. (ZIP) [file pone.0094198.s001.zip › Figures S1-20/Figure S9 1H-NMR of broussoside C.tiff]
